# Diversity of transcripts and transcript processing forms in plastids of the dinoflagellate alga *Karenia mikimotoi*

**DOI:** 10.1007/s11103-015-0408-9

**Published:** 2016-01-14

**Authors:** Richard G. Dorrell, George A. Hinksman, Christopher J. Howe

**Affiliations:** Department of Biochemistry, University of Cambridge, Cambridge, UK; School of Biology, École Normale Supérieure, Paris, France

**Keywords:** RNA processing, Haptophytes, Transcript processing, Endosymbiotic gene transfer, Chloroplast evolution

## Abstract

Plastids produce a vast diversity of transcripts. These include mature transcripts containing coding sequences, and their processing precursors, as well as transcripts that lack direct coding functions, such as antisense transcripts. Although plastid transcriptomes have been characterised for many plant species, less is known about the transcripts produced in other plastid lineages. We characterised the transcripts produced in the fucoxanthin-containing plastids of the dinoflagellate alga *Karenia mikimotoi*. This plastid lineage, acquired through tertiary endosymbiosis, utilises transcript processing pathways that are very different from those found in plants and green algae, including 3′ poly(U) tail addition, and extensive substitutional editing of transcript sequences. We have sequenced the plastid transcriptome of *K. mikimotoi,* and have detected evidence for divergent evolution of fucoxanthin plastid genomes. We have additionally characterised polycistronic and monocistronic transcripts from two plastid loci, *psbD*-*tRNA*^*Met*^-*ycf4* and *rpl36*-*rps13*-*rps11*. We find evidence for a range of transcripts produced from each locus that differ in terms of editing state, 5′ end cleavage position, and poly(U) tail addition. Finally, we identify antisense transcripts in *K. mikimotoi*, which appear to undergo different processing events from the corresponding sense transcripts. Overall, our study provides insights into the diversity of transcripts and processing intermediates found in plastid lineages across the eukaryotes.

**Electronic supplementary material:**

The online version of this article (doi:10.1007/s11103-015-0408-9) contains supplementary material, which is available to authorized users.

## Introduction

As a consequence of their endosymbiotic origin, chloroplasts and other plastid lineages retain their own genomes, which encode proteins and other factors essential for their function (Dorrell and Howe 2015; Green 2011). Understanding how these genomes are transcribed, and ultimately expressed, is fundamental to understanding plastid physiology. Much has been studied about the diversity and processing intermediates of plastid transcripts in plants and green algae (Stern et al. 2010). Initially, plastid genes in these lineages are cotranscribed, forming polycistronic transcripts. These polycistronic transcripts may undergo processing events, including cleavage into monocistronic mRNAs, and substitutional editing (Stern et al. 2010). In addition to coding transcripts, plant plastids produce non-coding transcripts. These include antisense transcripts, which are transcribed from promoters located on the template strand of plastid genes (Georg et al. 2010; Sharwood et al. 2011; Zghidi-Abouzid et al. 2011). The overaccumulation of plastid antisense transcripts is likely to be deleterious, as they anneal to and impede the expression of sense transcripts (Hotto et al. 2010; Zghidi-Abouzid et al. 2011). Accordingly, some plastid antisense transcripts are degraded during transcript processing (Hotto et al. 2015; Sharwood et al. 2011).

This study was designed to investigate the range of transcripts, and transcript processing events, in a plastid lineage that is evolutionarily distant to plants and green algae, that of the fucoxanthin-containing dinoflagellate *Karenia mikimotoi*. Dinoflagellates are an ecologically important group of algae. Some dinoflagellate species form photosynthetic symbioses within corals, while others may form toxic “red tides” (Dorrell and Howe 2015). The majority of photosynthetic dinoflagellates harbour plastids derived from red algae that contain the pigment peridinin. Other dinoflagellates have replaced the peridinin-containing plastids with plastids of different phylogenetic derivations. These include fucoxanthin-containing dinoflagellates such as *K. mikimotoi*, in which the original peridinin plastid has been replaced by one derived from haptophyte algae (Dorrell and Howe 2015).

The transcript processing events associated with dinoflagellate plastids have been characterised, and appear to be quite different from those observed in plants. Plastid transcripts in some peridinin-containing dinoflagellates undergo extensive substitutional sequence editing, which appears to have evolved independently from the editing observed in plant plastids (Knoop 2011; Mungpakdee et al. 2014; Zauner et al. 2004). Plastid transcripts in peridinin-containing dinoflagellates additionally receive a 3′ poly(U) tail (Wang and Morse 2006). This pathway is not found in the plastids of plants or other major eukaryotic algae, although it has been identified in the “chromerid” algae *Chromera velia* and *Vitrella brassicaformis*, which possess plastids related to the peridinin lineage (Dorrell and Howe 2012, 2015; Janouškovec et al. 2010). Remarkably, poly(U) tail addition and sequence editing also occur in fucoxanthin-containing dinoflagellate plastids, despite being absent from the plastids of free-living haptophytes (Dorrell and Howe 2012; Jackson et al. 2013). This indicates that these transcript processing pathways have been retained from the ancestral plastid symbioses, and applied to the incoming replacement lineage following the serial endosymbiotic event (Dorrell and Howe 2012, 2015).

Previously, we have conducted a genome-wide survey of the coding transcripts produced in the plastids of the fucoxanthin-containing dinoflagellate *Karlodinium veneficum* (Richardson et al. 2014), for which an entire plastid genome sequence is available (Gabrielsen et al. 2011). However, little is known about the plastid transcripts produced in other fucoxanthin-containing dinoflagellate species, or the range of processing forms and non-coding transcripts produced in fucoxanthin-containing plastids. In this study, a plastid transcriptome was generated for *K. mikimotoi,* from which a diverse range of polyuridylylated transcripts of probable plastid origin was identified, along with evidence for divergent evolution of individual fucoxanthin plastid genomes. In addition, the range of different transcripts produced from the *K. mikimotoi rpl36*-*rps13*-*rps11* and *psbD*-*tRNA*^*Met*^-*ycf4* loci was characterised in detail, and striking differences in the roles of transcript terminal cleavage, editing and poly(U) tail addition were found for each locus. Finally, antisense transcripts were identified for multiple loci in the *K. mikimotoi* plastid. These transcripts do not appear to undergo the same processing events as the corresponding sense transcripts. This study provides insights into the evolutionary history of fucoxanthin dinoflagellates, and the transcript processing events found in this unusual plastid lineage.

## Materials and methods

### Cultures and nucleic acid isolation

*Karenia mikimotoi* RCC1513 was grown in modified k/2 medium, at 20 °C, under an alternating 12 h: 12 h cycle of 50 μEm^−2^ s^−1^ light: dark, as previously described (Dorrell and Howe 2012). Cultures were harvested in early stationary phase (approximately 2 months after inoculation). Cells were pelleted and washed three times with sterile culture medium prior to the isolation of nucleic acids.

Total cellular RNA was isolated by phase extraction with Trizol reagent (Ambion), as previously described (Dorrell and Howe 2012). Residual DNA contamination was removed from RNA samples by treatment with RNase-free DNase (Roche), and cleaning with an RNeasy column (Qiagen), as previously described (Barbrook et al. 2012; Dorrell and Howe 2012). Each RNA sample was confirmed to be DNA-free by two rounds of direct PCR, using the RNA sample as the PCR template. Genomic DNA was isolated from cell pellets by phase extraction, as previously described (Barbrook et al. 2012). Nucleic acid concentrations were quantified using a nanodrop spectrophotometer.

### Generation and assembly of next generation sequencing products

Double-stranded cDNA was synthesised from 4 μg *Karenia mikimotoi* total cellular RNA using a Maxima H Minus synthesis kit (Thermo). The initial cDNA synthesis reaction was performed using an oligo-d(A) primer previously shown to anneal to polyuridylylated dinoflagellate plastid transcripts (Barbrook et al. 2012; Dorrell and Howe 2012), and the second strand was synthesised according to the manufacturer’s instructions. 0.5 μmol EDTA was added to stop the reaction, and products were cleaned with a MinElute spin column (Qiagen) using a guanidine thiocyanate binding buffer, and were eluted in Tris–EDTA buffer at pH 8.

Double stranded cDNA was quantified using a Qubit fluorometer (Invitrogen) according to the manufacturer’s instructions. A sequencing library was generated from 100 ng purified product using a NexteraXT tagmentation kit (Illumina). The library was sequenced over 500 cycles using a MiSeq sequencer. Reads were trimmed using the Miseq reporter version 2.0.26, and assembled into 287,906 contigs using ELAND (Illumina), Trinity (Haas et al. 2013) and GeneIOUS v.4736 (Kearse et al. 2012).

Sequences of potential plastid origin were identified by reciprocal BLAST searches against protein sequences, generated by conceptual translations of plastid genes, from the fucoxanthin-containing dinoflagellate *Karlodinium veneficum* (Gabrielsen et al. 2011; Richardson et al. 2014), the cultured haptophytes *Emiliania huxleyi, Chrysochromulina tobin, Phaeocystis globosa*, and *Pavlova lutheri* (Baurain et al. 2010; Hovde et al. 2014; Puerta et al. 2005), and the uncultured haptophyte C19847 (Cuvelier et al. 2010). Initially, a tBLASTn search was performed of the complete contig sequences using protein queries from all five species, using a threshold expect value of E-05. This expect value was used as it was previously found to be adequate to identify highly divergent genes in the plastid genome of *K. veneficum* (Richardson et al. 2014). We additionally tried repeating each BLAST search with greater expect values than E−05, but could not find any additional sequences in these searches of predicted fucoxanthin plastid origin.

581 contigs were identified through this approach that matched a query sequence with an expect value equal or lower to the threshold value. These contigs were then compared with the entire NCBI database using BLASTx. 271 contigs from within this set that recovered plastid or cyanobacterial sequences as the first hit were selected for further analysis.

Contigs that might correspond to nuclear genes for plastid-targeted proteins in *Karlodinium veneficum* were identified by tBLASTn searches of 17,434 *Karlodinium* EST sequences located on NCBI (Patron et al. 2006), and 208,375 transcript sequences located on the Marine Microbial Eukaryote Transcriptome Sequencing Project Database (Keeling et al. 2014), using the above protein sequences, as well as protein sequences generated by the conceptual translation of *Karenia mikimotoi* transcripts as queries, but excluding the protein sequences of genes previously shown to be located on the *Karlodinium* plastid genome (Gabrielsen et al. 2011; Richardson et al. 2014). From this, 5 NCBI EST sequences and 42 MMETSP transcript sequences were found that were likely to be derived from nucleus-encoded, plastid-targeted genes, and were assembled into contigs as above.

Transfer RNA sequences in each contig were identified using the ARAGORN web server (Laslett and Canback 2004). Plastid targeting sequences in nucleus-encoded *Karlodinium veneficum* proteins were predicted using SignalP v.3.0, ASAfind and ChloroP (Bendtsen et al. 2004; Gruber et al. 2015). Alternative translation initiation codons were identified using NCBI ORF Finder (Rombel et al. 2002).

### RT-PCR and Sanger sequencing

Reverse transcriptions for RT-PCR were performed of total cellular RNA from *K. mikimotoi* using a Superscript III kit (Invitrogen) as previously described (Dorrell et al. 2014). For circular RT-PCRs, total cellular RNA was circularised using T4 RNA ligase (New England Biolabs) as previously described (Barbrook et al. 2012; Dorrell and Howe 2012), and the ligation product was used directly as a template for RT-PCR. For RNA ligase-mediated 5′ RACE, 1 µg freshly harvested total cellular RNA was ligated to 1 μg of a custom synthesised RNA adapter sequence using 10 U Promega T4 RNA ligase, 6 μl Promega T4 10× buffer, 40 U RNAsin, 30 μl 40 % PEG and nuclease-free water to 60 μl at 16 °C for 16 h, and reverse transcriptions and PCR reactions were then performed as described elsewhere (Dang and Green 2010; Scotto-Lavino et al. 2006). PCR and thermal asymmetric interlaced PCR reactions were performed as previously described (Barbrook et al. 2012; Dorrell and Howe 2012; Takishita et al. 1999). Primer sequences corresponding to each experiment are given within the Supplementary Materials.

PCR products were visualised by electrophoresis in a 1 % agarose-TBE gel containing ethidium bromide. Oligo-d(A) and linear RT-PCR products were purified using a Qiaquick column kit (Qiagen), and directly sequenced using an Applied Biosystems 3730xl DNA Analyzer. Where multiple bands were detectable, individual products were separated by electrophoresis, cut out of the agarose gel, and purified as before. Circular RT-PCR and TAiL-PCR products were purified and directly ligated into pGEM-TEasy vector sequence (Promega), and introduced into competent *Escherichia coli* DH5α by transformation. Plasmids from individual colonies were purified using a GeneJET Miniprep kit (Thermo), and sequenced as before. Sequences were deposited in GenBank, under Accession Numbers KM065572-KM065732.

### Northern blotting

Northern blots were performed using *K. mikimotoi* total cellular RNA essentially as previously described (Dorrell et al. 2014). 3 μg total cellular RNA per blot was resuspended in 20 μl each of water and formamide, melted at 65 °C for 5 min, snap frozen, and separated by electrophoresis in an RNase-free 1 % TBE gel, containing 500 mg/l guanidine thiocyanate, at 100 V for 90 min. To confirm RNA integrity following electrophoresis, a separate lane, containing an additional 200 ng sample of total cellular RNA, was run on the same gel, stained after electrophoresis with ethidium bromide, and visualised with ultraviolet light (UV). RNA was transferred overnight to an RNase-free positively charged nitrocellulose membrane (Roche) according to the manufacturer’s instructions. After transfer, RNA was crosslinked to the membrane by exposure for 2 min in a 1200 μEm^−2^ s^−1^ UV transilluminator. The compressed gel slice from the transfer was stained with ethidium bromide and visualised with UV as before, to confirm that the RNA had not degraded during the transfer time period.

Blots were hybridised overnight at 65 °C with RNA probes, generated by transcription in vitro with a digoxigenin-labelling kit (Roche), according to the manufacturer’s instructions. Template sequences were generated by ligating PCR products corresponding to the 5′ ends of the *K. mikimotoi psbD, ycf4, rpl36, rps13* and *rps11* genes into pGEM-T Easy vector sequence (Promega), and amplifying the ligation products using a T7 primer and a PCR forward primer, to generate products containing the 49 bp T7 arm of the vector sequence fused to the insert. Northern blot probe sequences are given within the Supplementary Materials. Hybridisation products were visualised using an anti-digoxigenin/CPD-star system (Roche), per the manufacturer’s instructions.

## Results

### Oligo-d(A) cDNA sequencing reveals the polyuridylylated plastid transcriptome of *Karenia mikimotoi*

To characterise the diversity of polyuridylylated transcripts produced in plastids of the fucoxanthin-containing dinoflagellate *Karenia mikimotoi*, double stranded cDNA was generated from *Karenia mikimotoi* total cellular RNA using an oligo-d(A) cDNA synthesis primer, which anneals to dinoflagellate plastid poly(U) tails (Barbrook et al. 2012; Dorrell and Howe 2012). Illumina sequences were obtained using a MiSeq platform from the cDNA library, and were assembled into 287,106 contigs. 271 contigs of probable plastid origin were identified within this library by reciprocal BLAST searches against plastid sequences from the related fucoxanthin-containing dinoflagellate *Karlodinium veneficum* (Gabrielsen et al. 2011; Richardson et al. 2014), and five free-living haptophyte species (Baurain et al. 2010; Cuvelier et al. 2010; Hovde et al. 2014; Puerta et al. 2005). A PCR forward primer was designed against the 5′ end of the furthest upstream contig identified for each gene, and an individual RT-PCR was performed, using an oligo-d(A) cDNA synthesis and PCR reverse primer, and the PCR forward primer specific to the contig, to confirm that the contig (and all downstream contigs of the gene in question) formed part of a polyuridylylated transcript (Table S1).

**Fig. 1:**
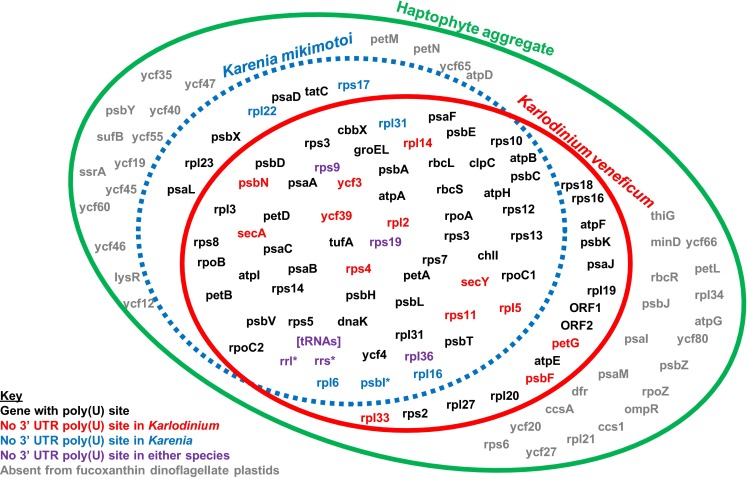
The *Karenia mikimotoi* plastid transcriptome. The *Venn diagram* shows the total polyuridylylated transcripts assigned to the *Karenia mikimotoi* plastid (*blue*, *dashed circle*), overlaid with the coding content of the plastid genome of the related fucoxanthin-containing dinoflagellate *Karlodinium veneficum* (*red circle*) (Gabrielsen et al. 2011). Other genes that are located in the plastids of other haptophyte species (*Emiliania huxleyi, Chrysochromulina tobin, Phaeocystis globosa, Pavlova lutheri,* and the uncultured species C19847 27,28), which we could not identify in the *Karenia mikimotoi* plastid and are not plastid-encoded in *Karlodinium veneficum*, are contained within the larger *green circle*. Genes are shaded according to their poly(U) tail addition state. Genes shaded in *black* possess a poly(U) site in the associated 3′ UTR in all fucoxanthin-containing dinoflagellate species in which they are plastid-located. Genes shaded in *blue*, *red*, and *purple* respectively lack associated poly(U) sites in their associated 3′ UTR in *Karenia mikimotoi*, in *Karlodinium veneficum*, and in both fucoxanthin-containing dinoflagellate species. These genes do not give rise to polyuridylylated monocistronic transcripts, although may form part of polyuridylylated polycistronic transcripts. Genes labelled with an *asterisk* (*psbI, rrl, rrs*) are those that could not be identified to give rise to polyuridylylated polycistronic or monocistronic transcripts through oligo-d(A) RT-PCR, but are inferred to be located in the plastid genome. Genes in *parentheses* were not identified from the assembly of next generation sequencing data, and were identified using alternative strategies (e.g. TAiL-PCR)

Through this pipeline, 65 protein-coding genes were identified that are probably located in the *Karenia mikimotoi* plastid (Fig. 1, genes in blue circle except those asterisked or in square brackets; Table S2). This is broadly similar to the situation for the *Karlodinium veneficum* plastid genome, which retains 73 protein-coding genes, but far fewer than the 110–115 protein-coding genes found in the plastid genomes of free living haptophytes (Gabrielsen et al. 2011; Puerta et al. 2005) (Fig. 1, genes in green circle).

### Distribution of poly(U) sites within the *Karenia mikimotoi* plastid

For 57 of the 65 genes confirmed to give rise to a polyuridylylated transcript by oligo-d(A) RT-PCR, the poly(U) site was located in the adjacent 3′ UTR (Fig. 1; genes in black and in red). In the remaining 8 cases, the oligo-d(A) RT-PCR generated a polycistronic product, containing the gene from which the PCR forward primer was designed, and one or more genes located downstream, with a poly(U) tail located in the 3′ UTR of the final downstream gene (Fig. 1; genes in purple and in blue, except those asterisked; Tables S2, S3).

Contigs that corresponded to a *psbI* transcript, and to regions of the 16S and 23S ribosomal RNAs were additionally identified within the next generation sequencing dataset (Fig. 1, asterisked genes). However, corresponding polyuridylylated transcripts could not be found by RT-PCR for any of these genes, even following a second round of PCR amplification, using the initial RT-PCR product for each gene as template. To determine whether these transcripts are produced within the *Karenia mikimotoi* plastid, the underlying genes were sequenced, and compared to the transcript sequence. Editing was inferred for each transcript sequence (Fig. S1). Editing is associated with plastid transcripts, but not nuclear transcripts in fucoxanthin-containing dinoflagellates, indicating that the genes for *psbI*, and the 16 and 23S rRNA subunits, are located within the *K. mikimotoi* plastid (Dorrell and Howe 2012; Jackson et al. 2013).

No predicted tRNA sequences were identified within the next generation sequencing dataset. To look for plastid genes that encode tRNAs, bidirectional thermal asymmetric interlaced PCR (TAiL-PCR) extensions were performed for a representative sample of plastid genes, using a genomic DNA template (Liu et al. 1995). These included five genes (*psbA, psbC, psbD, psaA, rbcL*) for which the underlying 3′ UTR sequences in *K. mikimotoi* have previously been obtained (Dorrell and Howe 2012; Takishita et al. 1999), as well as one representative multigene contig (*rpl36*-*rps13*-*rps11*) assembled directly from the next generation sequencing data, and the putative plastid *psbI* gene (Table S3). tRNA genes were identified adjacent to the *psbC, psbD, psbI* and *rbcL* genes (Table S3). To test whether these tRNA genes gave rise to polyuridylylyated transcripts, oligo-d(A) RT-PCRs were performed, using PCR primers specific to the sequence immediately 5′ end of each tRNA gene, as before (Table S1); however, no polyuridylylated transcripts were identified (Fig. 1; Tables S1, S3).

### Plastid-to-host gene transfer events in individual fucoxanthin-containing dinoflagellates

The plastid genome of the fucoxanthin-containing dinoflagellate *Karlodinium veneficum* retains far fewer genes than the plastid genomes of free-living haptophytes (Dorrell and Howe 2015; Gabrielsen et al. 2011). The genes that are plastid-located in haptophytes, but not retained on the fucoxanthin plastid genome, may have been relocated to the dinoflagellate nucleus since its endosymbiotic acquisition. Examples of gene transfer from the fucoxanthin-containing endosymbiont to the dinoflagellate host nucleus have been characterised (Burki et al. 2014; Ishida and Green 2002; Miller and Delwiche 2015). However, none of these putatively transferred genes corresponds to the genes that have been lost from the *Karlodinium veneficum* plastid genome, and the ultimate evolutionary fate of these latter genes remains unknown.

The *Karenia mikimotoi* plastid transcriptome provides evidence for independent plastid-to-host gene transfer events in individual fucoxanthin plastid lineages. For example, seven genes were identified to give rise to polyuridylylated transcripts in *Karenia mikimotoi* that are not known to be located on the *Karlodinium veneficum* plastid genome (Fig. 1). To determine the likely cellular location of these genes in *Karlodinium veneficum*, individual BLAST searches were performed for each gene using *Karlodinium veneficum* transcript sequences located on GenBank (Burki et al. 2014; Patron et al. 2006) and the Marine Microbial Eukaryote Transcriptome Sequencing Project (Keeling et al. 2014). Homologous sequences for five genes (*psaD, psaL, rpl22, rpl23*, and *tatC*) were identified (Table S4). Of these, the *psaD*, *rpl22* and *tatC* genes were found to encode proteins that contain a predicted N-terminal targeting sequence, consisting of a signal peptide, followed by an ASAFAP-type cleavage site, and a predicted plastid transit peptide, upstream of the conserved sequence region (Table S4). These are consistent in structure with plastid targeting sequences previously identified in fucoxanthin-containing dinoflagellates (Patron and Waller 2007; Yokoyama et al. 2011). Thus, the *psaD, rpl22* and *tatC* genes at least have been relocated from the *Karlodinium veneficum* plastid to the nucleus, following its divergence from *Karenia mikimotoi*.

### Post-endosymbiotic divergence in fucoxanthin plastid gene sequences

In addition to extensive gene loss, fucoxanthin plastid genomes are highly divergent in sequence organisation. Many of the genes in the *Karlodinium veneficum* plastid, for example, contain in-frame insertions or deletions not found in other plastid lineages (Gabrielsen et al. 2011; Richardson et al. 2014). Novel sequence insertions and deletions were likewise found in many of the *Karenia mikimotoi* sequences. To determine whether these insertions were conserved between both fucoxanthin-containing species, or evolved independently in each species, 9179 aa plastid protein sequence (from 54 plastid genes) from *Karenia mikimotoi* and *Karlodinium veneficum* were aligned against a reference set of proteins from the haptophytes *Emiliania huxleyi, Phaeocystis globosa* and *Pavlova lutheri* (Table S5). In total, 109 insertions and deletions were identified in fucoxanthin-containing species that are not present in free-living haptophytes (Table S5). Of these, only 10 are conserved between both fucoxanthin-containing dinoflagellates, while the remaining 99 are unique to either *Karenia mikimotoi* or to *Karlodinium veneficum* (Table S5). Thus, the plastid genomes of fucoxanthin-containing dinoflagellates have diverged substantially in coding sequence content since their endosymbiotic acquisition.

The *Karlodinium veneficum* plastid has previously been proposed to utilise ATT as an alternative translation initiation codon, in addition to ATG (Gabrielsen et al. 2011). Several of the *Karenia mikimotoi* plastid gene sequences amplified by TAiL-PCR, such as *psaA, rpl36,* and *ycf4* were found not to possess a conventional ATG initiation codon (Fig. S2). In each case, an in-frame translation termination codon was identified in the 5′ UTR sequence immediately upstream of the conserved coding region, ruling out the possibility that translation is initiated from ATG codons located further upstream (Fig. S2). The 5′ ends of the *psaA, rpl36* and *ycf4* transcripts were sequenced by RT-PCR, using RNA circularised with T4 RNA ligase (Table S6), and confirmed not to contain ATG codons introduced by editing of the transcript sequence (Fig. S2).

Instead, each transcript possessed a predicted alternative translation initiation codon at the 5′ end of the conserved coding region. *ycf4* appears to use an ATT codon positioned immediately downstream of the in-frame stop codon, similar to the alternative initiation codons identified in *Karlodinium veneficum* (Fig. S2, panel A) (Gabrielsen et al. 2011). In contrast, translation of the *psaA* gene is predicted to be initiated from a TTG codon, and translation of *rpl36* from a GTG codon (Fig. S2, panel B). Neither TTG nor GTG has previously been reported to function as an alternative initiation codon in any fucoxanthin-containing dinoflagellate plastid, including in *Karlodinium veneficum* (Gabrielsen et al. 2011). Thus, the *Karenia mikimotoi* plastid has diverged from *Karlodinium veneficum* in terms of the range of variant initiation codons used.

### Diversity of transcripts produced from two plastid loci

Previous studies of transcripts in the plastids of peridinin-containing dinoflagellates and their closest relatives have identified a diverse range of processing events. In addition to transcripts containing a 3′ poly(U) tail, many plastid genes in these lineages also give rise to non-polyuridylylated transcripts (Barbrook et al. 2012; Dorrell et al. 2014; Nelson et al. 2007). In addition, dinoflagellates have been shown to give rise to polyuridylylated polycistronic transcripts, which for certain loci may be highly abundant (Dang and Green 2010; Janouškovec et al. 2013; Nisbet et al. 2008). Finally, different transcripts within dinoflagellate plastids may vary in editing state. For example, previous studies of peridinin dinoflagellates (Dang and Green 2009) and of the *Karenia mikimotoi* plastid (Dorrell and Howe 2012) have shown that transcripts that terminate in a 3′ poly(U) tail are typically more extensively edited than transcripts that extend downstream of the poly(U) site.

We wished to characterise the diversity of transcript processing forms found in the *K. mikimotoi* plastid. In particular, we wished to determine to what extent non-polyuridylylated, polycistronic, and partially edited transcripts might form a component of the *K. mikimotoi* plastid transcriptome. As resolution of the 5′ and 3′ terminal positions of individual transcripts (Park et al. 2014) and discrimination of very low level processing events from experimental artefacts (Guo et al. 2015; Lin et al. 2015) are difficult to perform with transcriptomic surveys of untreated RNA, we chose to supplement our initial transcriptomic data with detailed experimental characterisation of transcript diversity at two multigene plastid loci. The *rpl36*-*rps13*-*rps11* and *psbD*-*tRNA*^*Met*^-*ycf4* loci were selected as representatives for more detailed study. The *rps13, rps11,psbD* and *ycf4* genes possess associated 3′ UTR poly(U) sites, while the *rpl36* and *tRNA*^*Met*^ genes do not (Fig. 1).

**Fig. 2:**
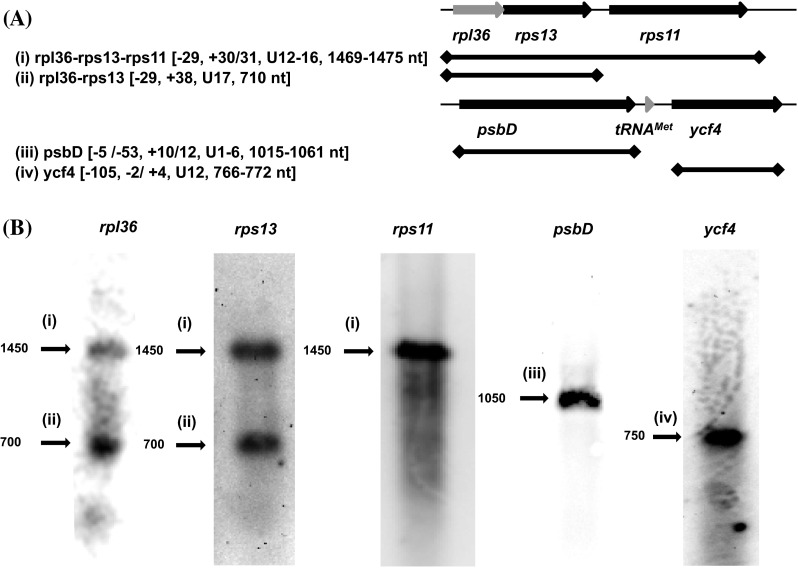
Diversity of plastid transcripts from the *rpl36*-*rps13*-*rps11* and *psbD*-*tRNA* ^*Met*^-*ycf4* loci. **a** Schematic diagrams of the *rpl36*-*rps13*-*rps11* and *psbD*-*tRNA* ^*Met*^-*ycf4* loci. Genes that possess associated poly(U) sites are shown in *black*, and genes that lack poly(U) sites in their associated 3′ UTR are shown in *grey*. *Thin black lines* correspond to non-coding DNA. The transcripts identified by circular RT-PCR that correspond to visible bands in each northern blot are shown for each locus. *Labels in parentheses* to the *left* of each transcript name correspond to the *labels above each band* in the northern blot, and *labels in square brackets* give the 5′ end position, 3′ end position, poly(U) tail length, and lengths of the transcripts identified. **b** The results of northern blots to identify transcripts covering *rpl36, rps13, rps11, psbD* and *ycf4*. Each band is labelled with the expected size of the corresponding transcript, as calculated by comparison to a DIG-labelled RNA molecular weight marker run on the same RNA gel

First, the diversity of transcript terminal positions associated with each locus was characterised by circular RT-PCR (Table S6). cDNA was synthesised from circularised RNA using primers specific to the *rps13, rps11, psbD* and *ycf4* genes (Fig. 2a). Each cDNA sample was then amplified using a range of PCR primers designed to anneal to different regions of the *rpl36*-*rps13*-*rps11* and *psbD*-*tRNA*^*Met*^-*ycf4* loci. For example, for *psbD* cDNA, PCRs were performed using two reverse primers designed to anneal to the *psbD* CDS, and ten forward primers, of which three were designed to anneal within *psbD* to detect monocistronic transcripts, two were designed within the intergenic region containing *tRNA*^*Met*^, and five were designed to anneal within *ycf4* to detect polycistronic transcripts covering all three genes (Table S6). Each possible combination of PCR reverse and forward primer (e.g. for *psbD,* 20 different combinations) was tested; each RT-PCR was repeated three times, using cDNA templates generated from independently isolated and circularised RNA samples; and for each gene, a minimum of twenty unique transcripts were cloned and sequenced (Table S7). To determine the most abundant transcripts produced from each locus, northern blots of *K. mikimotoi* RNA were hybridised with probes specific to *rpl36, rps13, rps11, psbD* and *ycf4* (Fig. 2b; Table S8).

### Terminal processing of transcripts from the *rpl36*-*rps13*-*rps11* locus

Through circular RT-PCR, polycistronic *rpl36*-*rps13*-*rps11* transcripts of 1450 nt length, and *rpl36*-*rps13* transcripts of approximately 700 nt length were identified [Fig. 2a; transcripts labelled (i) and (ii)]. These transcripts correspond in size to bands visible in the *rpl36* and *rps13* northern blot (Fig. 2b). Monocistronic *rps13* transcripts could not be identified via circular RT-PCR (Table S7), and no other significant hybridisation that might correspond to monocistronic transcripts was observed in either the *rpl36* or *rps13* northern blots (Fig. 2b). Although monocistronic *rps11* transcripts (of approx. 700–800 nt length) were identified through circular RT-PCR, bands corresponding to these transcripts were not detected in the *rps11* northern blot (Fig. 2; Table S7). Instead, the only significant hybridisation in the *rps11* blot was a band corresponding to the 1450 nt polycistronic *rpl36*-*rps13*-*rps11* transcripts (Fig. 2b). Thus, the most abundant transcripts produced from the *rpl36*-*rps13*-*rps11* locus are polycistronic.

Sixteen of the seventeen *rpl36*-*rps13*-*rps11* transcripts identified by circular RT-PCR contained a 3′ poly(U) tail (Table S7). Although one non-polyuridylylated *rpl36*-*rps13*-*rps11* transcript was identified by circular RT-PCR, this transcript was only 1118 nt long (Table S7), and thus could not correspond to the predominant hybridisation found in the *rps11* northern blot (Fig. 2). In contrast, only nine of the thirty *rpl36*-*rps13* transcripts were polyuridylylated (Table S7). Of the remaining twenty-one *rpl36*-*rps13* transcripts, eleven were between 650 and 800 nt length, corresponding in size to the *rpl36*-*rps13* transcript hybridisation in the *rpl36* and *rps13* northern blots, and contained complete *rpl36* and *rps13* open reading frames, i.e. may be highly abundant and translationally competent (Fig. 2b; Table S7). Thus, while the overwhelming majority of the *rpl36*-*rps13*-*rps11* transcripts receive poly(U) tails, a significant proportion of *rpl36*-*rps13* transcripts may not.

The 3′ ends of the *rpl36*-*rps13*-*rps11* transcripts were highly uniform, terminating in all but two cases at the consensus *rps11* poly(U) site, which is positioned 30 nt downstream of the *rps11* translation termination codon. In contrast, the 3′ ends of the *rpl36*-*rps13* transcripts were heterogeneous. Several of the *rpl36*-*rps13* transcripts, including three polyuridylylated transcripts, extended at the 3′ end into the *rps11* CDS (Table S7). Notably, twenty-one of the *rpl36*-*rps13* transcripts, and all but one *rpl36*-*rps13*-*rps11* transcript, terminated at the 5′ end between 25 nt and 29 nt upstream of the *rpl36* CDS (Fig. 2a; Table S7). Thus, the majority of transcripts produced from the *rpl36*-*rps13*-*rps11* locus undergo similar 5′ end processing events.

### Terminal processing of transcripts from the *psbD*-*tRNA*^*Met*^-*ycf4* locus

For both *psbD* and *ycf4*, evidence was found for highly abundant monocistronic transcripts. The *psbD* northern blot yielded a single band, corresponding to transcripts of 1050 nt length [Fig. 2b; hybridisation labelled (iii)]. For the *ycf4* northern blot, a single band at 750 nt was observed [Fig. 2b; band labelled (iv)]. Monocistronic transcripts, of equivalent sizes to these bands, were obtained in each corresponding circular RT-PCR (Fig. 2a; Table S7). A small number of polycistronic *psbD*-*tRNA*^*Met*^*, tRNA*^*Met*^*-ycf4,* and *psbD*-*tRNA*^*Met*^-*ycf4* transcripts were additionally identified through circular RT-PCR (Table S7). However, none of these transcripts was of a size that corresponded to significant hybridisation in the *psbD* or *ycf4* blots, suggesting that they are low in abundance (Fig. 2b). Thus, for the *psbD*-*tRNA*^*Met*^-*ycf4* locus, the most abundant transcripts are monocistronic, with polycistronic transcripts forming only a small proportion of the total transcript pool.

For both *psbD* and *ycf4*, both polyuridylylated and non-polyuridylylated monocistronic transcripts were sequenced (Table S7). None of the polycistronic transcripts identified for this locus was polyuridylylated (Table S7). All of the polyuridylylated *ycf4* transcripts were of a size corresponding to the hybridisation observed in the northern blot, but none of the non-polyuridylylated transcripts was of this size (Fig. 2b; Table S7), suggesting that the majority of *ycf4* transcripts possess poly(U) tails. Similarly, six of the eight *psbD* transcripts sequenced that corresponded in size to hybridisation in the *psbD* northern blot, and contained a complete *psbD* open reading frame, possessed poly(U) tails (Fig. 2b; Table S7). Thus, the majority of translationally competent transcripts produced from the *psbD*-*tRNA*^*Met*^-*ycf4* locus possess poly(U) tails.

### Editing of plastid transcripts

We wished to characterise the editing states associated with different transcripts from the *rpl36*-*rps13*-*rps11* and *psbD*-*tRNA*^*Met*^-*ycf4* loci, and determine whether there are differences between the editing states associated with polyuridylylated versus non-polyuridylylated, and polycistronic versus monocistronic transcripts. To do this, the complete sequences of polyuridylylated *rpl36*-*rps13, rpl36*-*rps13*-*rps11, rps11, psbD* and *ycf4* transcripts were generated by assembling the oligo-d(A) RT-PCR products corresponding to each transcript, and the terminal regions of polyuridylylated transcripts identified by circular RT-PCR (Tables S3, S7). Each transcript was then resequenced twice, using PCR primers designed against the 5′ ends of each transcript, and oligo-d(A) primed cDNA synthesised from independently isolated RNA samples (Table S9). Editing events were found at between 2.3 and 6.3 % of the residues for each CDS (Table 1).

**Table 1:** An overview of the editing events identified on polyuridylylated and non-polyuridylylated transcripts covering different regions of the *rpl36-rps13-rps11* and *psbD-tRNA* ^*Met*^ *-ycf4* loci

1. rpl36-rps13-rps11			Transcript sequence					
Region	Length (bp)	Editing	rpl36-rps13	rpl36-rps13	rpl36-rps13-rps11	rpl36-rps13-rps11	rps11	rps11
			poly(U)	non-poly(U)	poly(U)	non-poly(U)	poly(U)	non-poly(U)
rpl36	164	Total	9	9	9	3	n.d.	n.d.
		%	5.49	5.49	5.49	1.83	n.d.	n.d.
rps13	462	Total	29	29	29	18	n.d.	n.d.
		%	6.28	6.28	6.28	3.90	n.d.	n.d.
Intergenic	43	Total	n.d.	0	0	0	n.d.	0
		%	n.d.	0.00	0.00	0.00	n.d.	0.00
rps11	732	Total	n.d.	n.d.	30	4	30	4
		%	n.d.	n.d.	4.10	0.55	4.10	0.55

2. psbD-tRNA^Met^-ycf4			Transcript sequence					
Region	Length (bp)	Editing	psbD	psbD	psbD-tRNA^Met^-ycf4	psbD-tRNA^Met^-ycf4	ycf4	ycf4
			poly(U)	non-poly(U)	poly(U)	non-poly(U)	poly(U)	non-poly(U)
5′ UTR	132	Total	3	0	n.d.	n.d.	n.d.	n.d.
		%	2.27	0.00	n.d.	n.d.	n.d.	n.d.
psbD	999	Total	22	17	13	9	n.d.	n.d.
		%	2.20	1.70	1.30	0.90	n.d.	n.d.
tRNA^Met^/intergenic	262	Total	n.d.	0	0	0	n.d.	0
		%	n.d.	0.00	0.00	0.00	n.d.	0.00
ycf4	664	Total	n.d.	n.d.	0	0	37	11
		%	n.d.	n.d.	0.00	0.00	6.07	1.80

"n.d." indicates that the transcript in question did not cover the corresponding region of sequence

To determine whether the polyuridylylated *rpl36*-*rps13, rpl36*-*rps13*-*rps11, rps11, psbD* and *ycf4* transcripts are more highly edited than non-polyuridylylated equivalents, cDNA was synthesised using primers positioned downstream of the *psbD, ycf4, rps13* and *rps11* poly(U) sites (Table S9). RT-PCR was performed using the same PCR forward primers as used for oligo-d(A) primed RT-PCR (Table S8). As before, each RT-PCR was performed three times using independently isolated RNA samples, and the consensus sequence of each transcript was assembled from the RT-PCR products obtained and, where possible, the terminal regions of transcripts that extended through each poly(U) site as obtained by circular RT-PCR. Consistent with previous data, the transcripts that extended through the *rps11, psbD* and *ycf4* poly(U) sites were less extensively edited than their polyuridylylated equivalents (Dorrell and Howe 2012). No editing events were found on any non-polyuridylylated transcript that were not also found in the corresponding polyuridylylated transcript. Most dramatically, only four of the thirty editing sites (13.3 %) within the *rps11* CDS that were found on polyuridylylated transcripts were also edited on transcripts that extended through the *rps11* poly(U) site (Table 1). In contrast, the non-polyuridylylated *rps13* RT-PCR sequences were edited to the same extent as polyuridylylated *rpl36*-*rps13* transcripts (Table 1, S7). Thus, unlike the situation for *rps11, psbD* and *ycf4*, processing of the *rps13* poly(U) site is not correlated with transcript editing.

To determine whether there were differences in the editing events associated with polycistronic versus monocistronic transcripts, the sequences for polycistronic *rpl36*-*rps13*-*rps11* and *psbD*-*tRNA*^*Met*^-*ycf4* transcripts were compared to those of monocistronic *rps11,psbD* and *ycf4*, and dicistronic *rpl36*-*rps13* transcripts. Sequences for polycistronic *psbD*-*tRNA*^*Met*^-*ycf4* transcripts were generated using a PCR forward primer positioned within *psbD,* and a PCR reverse primer positioned within *ycf4* (Table S9). Sequences were amplified from both oligo-d(A) cDNA (corresponding to polyuridylylated transcripts) and cDNA generated using the *ycf4* 3′ UTR cDNA primer (corresponding to non-polyuridylylated transcripts); as before, each RT-PCR was performed three times, using independently isolated RNA samples.

The polyuridylylated *rpl36*-*rps13*-*rps11* transcripts were edited to completion, containing every editing event found on the *rpl36*-*rps13* and *rps11* transcripts (Table 1). In contrast, the *psbD*-*tRNA*^*Met*^-*ycf4* transcript sequences were less extensively edited than the monocistronic *psbD* or *ycf4* transcripts (Table 1). Surprisingly, no editing was detected within the *ycf4* CDS of polycistronic *psbD*-*tRNA*^*Met*^-*ycf4* transcripts, even for the transcript amplified from oligo-d(A) cDNA (Table 1). Thus, editing of *ycf4* is specifically associated with monocistronic transcripts, with polycistronic transcripts not undergoing editing of *ycf4* even if they are polyuridylylated.

### Antisense transcripts are present in fucoxanthin-containing plastids

**Fig. 3:**
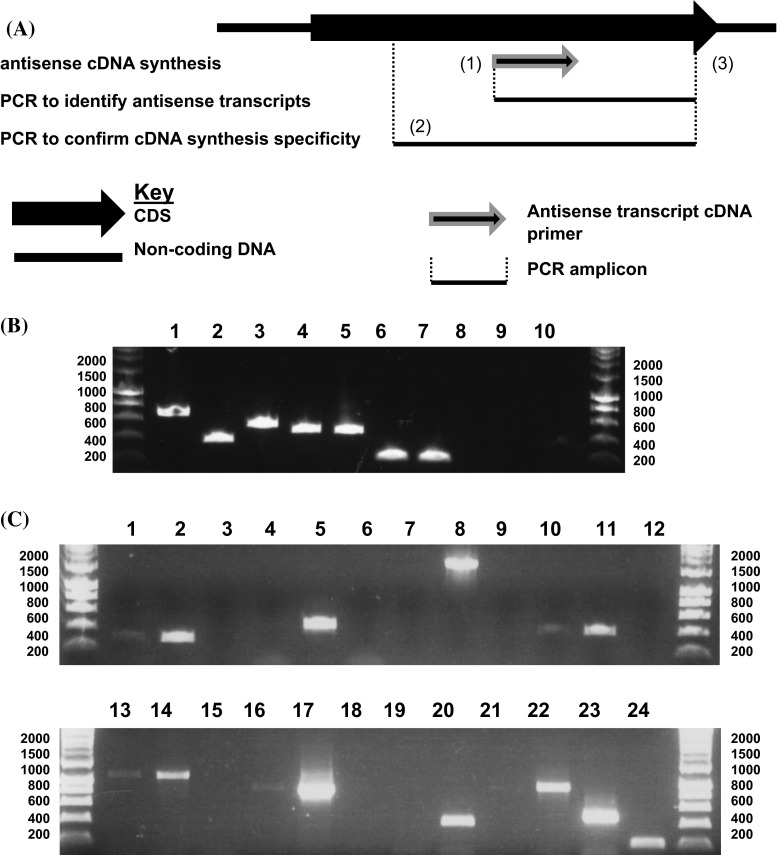
Presence of antisense transcripts in fucoxanthin-containing dinoflagellate plastids. **a** A diagram of the RT-PCRs used to detect antisense transcripts in the *K. mikimotoi* plastid. A primer with the same sequence as the non-template strand of the CDS (primer 1) was used to synthesise cDNA from antisense transcripts, and PCRs were performed on this template using the cDNA synthesis primer, and a complementary primer positioned downstream of the cDNA synthesis site (primer 2). A separate PCR was performed in each case, using the same cDNA template, and PCR primers flanking the cDNA synthesis site (primers 2, 3), to test for possible promiscuous annealing of the cDNA synthesis primer to sense transcripts. **b** A gel photograph of the RT-PCRs performed. Hyperladder I (Bioline) was used as a size marker, with the positions of representative size bands given at each side of the photo. *Lanes 1–7* RT-PCRs of antisense transcripts of seven plastid genes (*psbA, psbD, psaA, rbcL, ycf4, rps13, rps11*). *Lanes 8–10* cDNA template negative controls for antisense *psbA, ycf4* and *rps11* transcripts. **c** The results of RT-PCRs to confirm specificity of cDNA synthesis. *Lanes* *1–3* PCR using primers flanking the predicted *psbA* antisense cDNA synthesis site, and antisense (*1*) and sense (*2*) cDNA templates, and template negative conditions (*3*). *Lanes* *4–6* the same reactions, for *psbD*; *7–9* *psaA*, *10–12* *rbcL*, *13–15* *ycf4*, *16–18* *rps13*, *19–21* *rps11*. *Lanes 22–24* RT-PCRs for antisense *psbA, ycf4, rps11* transcripts using PCR primers positioned downstream of the cDNA synthesis site, as in **b** (antisense transcript positive controls)

We wished to determine whether antisense transcripts, similar to those previously identified in plant plastids, were present in the plastids of fucoxanthin-containing dinoflagellates (Georg et al. 2010; Hotto et al. 2010). To do this, a series of RT-PCRs to detect antisense transcripts were performed for seven *Karenia mikimotoi* plastid genes (*psbA, psbD, psaA, rbcL, rps13, rps11, ycf4*) (Fig. 3). For each RT-PCR, a cDNA synthesis primer was designed with the same sequence as the non-template strand of the gene (Fig. 3a, primer 1; Table S10). Each cDNA synthesis primer was confirmed by BLAST not to be similar to any sequence identified on the template strand of the corresponding gene, thus should preferentially anneal to antisense transcripts. PCRs were then performed using cDNA generated with each synthesis primer, and PCR primers positioned within each gene, downstream of the cDNA synthesis site (Fig. 3a; PCR amplicon bound by primers 1, 3).

For every gene tested, products were identified (Fig. 3b). To confirm that these products corresponded to antisense transcripts (rather than the result of the cDNA synthesis primer annealing promiscuously to sense transcripts), an additional PCR was performed for each gene, using the same cDNA template previously used to amplify antisense transcripts, and a PCR forward primer positioned upstream of the antisense transcript cDNA synthesis site (Fig. 3a; PCR amplicon bound by primers 2, 3). If the cDNA synthesis primer had promiscuously annealed to sense transcripts, products would be detected, whereas products would not be detected if the cDNA primer were specific to antisense transcripts (Fig. 3a). For several genes, no products were detected in these reactions (e.g. *psbD, psaA, rps11*; Fig. 3c; lanes 4, 7, 19), indicating that the cDNA primers used were entirely specific to antisense transcripts. For some genes, faint products were detected (e.g. *psbA, ycf4*; Fig. 3c; lanes 1, 13). To test whether these products formed a significant proportion of total PCR amplification, control PCR reactions were run for each gene using the same combination of PCR primers, and cDNA synthesised with a primer similar to the template strand of the gene (which would anneal to sense transcripts), and using the same cDNA preparations, and the PCR primers positioned downstream of the cDNA synthesis site previously used to identify antisense transcripts (Table S10). The PCR products identified in the control reactions were much more abundant than those generated with the antisense cDNA primer and sense transcript PCR primers (Fig. 3c; compare lanes 1, 2, 22; and lanes 13, 14, 23). Thus, promiscuous annealing is likely to only generate only a small proportion of the highly abundant products visible in Fig. 3a; while the majority correspond to plastid antisense transcripts.

To obtain independent evidence for the presence of antisense transcripts in the *K. mikimotoi* plastid, the 5′ ends of antisense transcripts were cloned using RNA ligase-mediated 5′ RACE, a technique that enables the amplification of transcript 5′ ends via the ligation of an RNA adapter (Fig. S3, panel A) (Dang and Green 2010; Scotto-Lavino et al. 2006). Two combinations of cDNA synthesis and PCR primers, designed to amplify specifically the 5′ ends of antisense transcripts, were designed for each of the *psbD, ycf4, rps13* and *rps11* genes (Table S11). Products were amplified using this approach for the *psbD, ycf4,* and *rps11* genes that terminated in a 5′ end adaptor ligation site (Fig. S3, panel B; Table S11; Table S12, panel A). None of the adaptor ligation sites for the transcripts amplified corresponded to regions of genomic sequence similar to either adaptor PCR primer, and similar products could not be identified in control 5′ RACE reactions performed without T4 RNA ligase, indicating that these products were not the result of promiscuous hybridisation of the adaptor PCR primers to *cis*-encoded sequence in each gene (Fig. S3, panel B). Thus, these sequences correspond specifically to the 5′ termini of plastid antisense transcripts.

### Strand-specific transcript processing events in fucoxanthin-containing dinoflagellates

In plant plastids, antisense and sense transcripts typically undergo different terminal processing events (Georg et al. 2010; Hotto et al. 2015), some of which may be linked to the preferential degradation of antisense transcripts (Sharwood et al. 2011). Previously, we have shown that some translationally non-functional transcripts in dinoflagellate plastids, such as those of pseudogenes, do not receive poly(U) tails or undergo significant levels of sequence editing, and undergo different terminal cleavage events from the transcripts of translationally competent paralogues (Dorrell et al. 2014; Richardson et al. 2014).

We wished to determine whether antisense transcripts in the *Karenia mikimotoi* plastid undergo different processing events from those associated with sense transcripts. To determine whether sense and antisense transcripts undergo different cleavage events, circular RT-PCRs were performed specific to antisense transcripts for the *psbD, ycf4, rps13* and *rps11* genes. The cDNA synthesis primers previously used to identify antisense transcripts of each gene were used, along with the same combinations of PCR primers used for circular RT-PCRs of sense transcripts at the *psbD*-*tRNA*^*Met*^-*ycf4* and *rpl36*-*rps13*-*rps11* loci (Tables S6, S10).

A range of transcripts were sequenced through this approach (Table S12, panel B). None contained regions of sequence similar to the complemented sequence of the cDNA primers employed, indicating that they were not generated by the promiscuous annealing of the cDNA synthesis primer to sense transcripts. For both the *rpl36*-*rps13*-*rps11* and *psbD*-*tRNA*^*Met*^-*ycf4* loci, transcripts were identified that either terminated at either end within the CDS, as well as antisense transcripts that extended through residues complementary to the sense transcript poly(U) sites previously identified by oligo-d(A) and circular RT-PCR (Table S12). However, no antisense transcripts were found either through circular RT-PCR or 5′ RACE that terminated at positions complementary to the consensus poly(U) sites for each gene, or the consensus 5′ end position associated with sense strand transcripts from the *rpl36*-*rps13*-*rps11* locus. Thus, sense and antisense transcripts are likely to undergo different terminal cleavage events. Although some of the antisense transcripts cloned contained differences from the underlying genomic sequence, consistent with the presence of editing, most of the antisense transcripts were not extensively edited: for example, of the 16 antisense *psbD, ycf4* and *rps11* transcript sequences cloned through 5′ RACE, only one was found to be edited on more than 1 % of the residues (Table S12). Thus, antisense transcripts are likely to undergo only limited editing events (Table S12).

To test for the presence of poly(U) tails on antisense transcripts, oligo-d(A) RT-PCRs were performed for antisense transcripts from each gene identified to localise to the *K. mikimotoi* plastid (Fig. S4). PCRs were performed using an oligo-d(A) primed cDNA template followed by PCR with oligo-d(A) and a primer with the same sequence as the template strand of each gene (Table S1). Each RT-PCR was repeated four times, using oligo-d(A) cDNA synthesised from independently isolated RNA samples.

For the majority of the genes tested, bands consistent with polyuridylylated antisense transcripts could not be amplified (Fig. S4). Polyuridylylated sense *psbA, ycf4* and *rps11* transcripts could be amplified from each of the cDNA templates using the same oligo-d(A) cDNA template as before, and a PCR primer with the same sequence as the non-template strand of each gene, confirming that the oligo-d(A) cDNA synthesis reaction had been successful (Fig. S4; Table S13). In total, only 16 of the 68 reverse primers tested yielded products (Fig. S4). This is significantly lower than the number of poly(U) sites detected for these genes by oligo-d(A) RT-PCRs for sense transcripts (Chi-squared, *P* < E^−12^). In addition, no polycistronic antisense transcripts were detectable by oligo-d(A) RT-PCR (Fig. S4; Table S3). Thus, poly(U) tail addition is preferentially associated with sense transcripts in the *K. mikimotoi* plastid.

## Discussion

We have investigated the diversity of plastid transcripts and transcript processing events in the fucoxanthin-containing dinoflagellate *Karenia mikimotoi*. We have generated a polyuridylylated plastid transcriptome for *Karenia mikimotoi*. The *Karenia mikimotoi* plastid differs significantly from its closest studied relative, the plastids of the related fucoxanthin-containing dinoflagellate *Karlodinium veneficum* (Gabrielsen et al. 2011). For example, over one in ten of the 68 genes assigned to the *Karenia mikimotoi* plastid have been lost from the *Karlodinium veneficum* plastid genome (Fig. 1). The differences in gene insertions and deletions, and the range of alternative translation initiation codons used by *Karenia mikimotoi* and *Karlodinium veneficum* are likewise consistent with divergent evolution of fucoxanthin-containing plastids (Fig. S2; Tables S4, S5).

We have additionally characterised the diversity of transcripts produced from two loci in *K. mikimotoi,rpl36*-*rps13*-*rps11* and *psbD*-*tRNA*^*Met*^-*ycf4*. The predominant transcripts generated from the *psbD*-*tRNA*^*Met*^-*ycf4* locus are monocistronic, whereas the most abundant transcripts produced from the *rpl36*-*rps13*-*rps11* locus are polycistronic (Fig. 2). Several of the *rpl36*-*rps13* transcripts extend into the 5′ end of the *rps11* CDS, indicating that they may be generated by alternative end processing of longer polycistronic transcripts covering all three genes, similar to what has previously been proposed to occur in fucoxanthin-containing and peridinin-containing dinoflagellates (Barbrook et al. 2012; Richardson et al. 2014), and in plant plastids (Rock et al. 1987). For the *psbD, ycf4* and *rps13* genes, non-polyuridylylated transcripts were identified that extended through the poly(U) site, and in the case of *rps13* these formed a significant component of the total transcripts present (Fig. 2; Table S7). Notably, the vast majority of transcripts produced from the *rpl36*-*rps13*-*rps11* locus, regardless of whether they are polyuridylylated downstream of *rps13*, *rps11*, or do not possess a poly(U) tail at all, utilise a single 5′ end processing site (Table S7).

The transcripts identified for the *rpl36*-*rps13*-*rps11* and *psbD*-*tRNA*^*Met*^-*ycf4* loci undergo distinctive patterns of editing. For *rps11* and *psbD*, transcripts that terminate in a poly(U) tail are more highly edited than transcripts that extend through the poly(U) site (Table 1). This may be due to the greater stability of polyuridylylated transcripts in fucoxanthin-containing plastids, or may alternatively suggest that poly(U) tail addition occurs concurrent to the completion of plastid transcript editing, as has previously been suggested to occur in peridinin dinoflagellates (Dang and Green 2009). More complex patterns of editing are observed within the *ycf4* CDS. For example, polycistronic *psbD*-*tRNA*^*Met*^-*ycf4* transcripts are not edited within *ycf4*, even if the transcript possesses a poly(U) tail, suggesting that cleavage of the 5′ end is additionally associated with the completion of editing (Table 1). It remains to be determined why transcripts produced from the *rpl36*-*rps13*-*rps11* and *psbD*-*tRNA*^*Met*^-*ycf4* loci undergo such different editing and cleavage events. This may be due to the functions of the proteins encoded by each locus, the presence of the tRNA sequence in *psbD*-*tRNA*^*Met*^-*ycf4*, or another reason entirely.

Finally, we have demonstrated the presence of transcripts containing antisense sequences to *K. mikimotoi* plastid genes (Figs. 3, S3). It remains to be determined how these antisense transcripts are generated. It is possible that these transcripts are not produced within the plastid itself, but instead are generated from regions of plastid sequence that have been relocated to the nucleus (NUPTs) (Lloyd and Timmis 2011; Smith et al. 2011). However, the antisense transcripts were identified by circular RT-PCR and RNA-ligase mediated 5′ RACE of a native, undigested RNA template (Fig. S3; Table S10). This should not be possible for nuclear transcripts, as the 5′ guanosine cap added during nuclear transcript processing inhibits transcript ligation by the T4 RNA ligase used for these experiments (Barbrook et al. 2012; Dang and Green 2010). In addition, none of the antisense transcripts contained transcript processing features associated with nuclear gene expression in eukaryotes (e.g. 3′ poly(A) tail addition) or specifically dinoflagellates (e.g. the presence of a 5′ spliced leader sequence) (Zhang et al. 2007). Alternatively, the antisense transcripts might be generated by self-priming and extension of plastid sense transcripts in vivo by an RNA-dependent RNA polymerase, which has been suggested to be present in plants (Zandueta-Criado and Bock 2004). However, this explanation is inconsistent with the absence of editing, complementary terminus positions, or other processing features that should be present if the antisense transcripts were generated from a mature sense transcript template (Hotto et al. 2015). Thus, it is likely that the antisense transcripts are generated by the transcription of the template strand of plastid genes. Antisense transcripts have previously been reported in plant plastids (Georg et al. 2010; Hotto et al. 2010), as well as in cyanobacteria (Sakurai et al. 2012), and the non-photosynthetic plastids of apicomplexan parasites (Bahl et al. 2010). However, to our knowledge, antisense transcripts have not previously been reported in an algal plastid lineage.

Notably, antisense transcripts in fucoxanthin-containing plastids appear typically not to receive poly(U) tails (Fig. S4). Previously, we and others have shown that poly(U) tail presence is correlated with high levels of transcript abundance in the plastids of chromerid algae (Dorrell et al. 2014; Janouškovec et al. 2013), and is associated with translationally competent transcripts rather than pseudogene and non-coding transcripts in chromerids and in *Karlodinium veneficum* (Dorrell et al. 2014; Richardson et al. 2014). If antisense transcript accumulation is indeed deleterious, the preferential application of poly(U) tails in fucoxanthin-containing dinoflagellate plastids to sense transcripts might enable them to be distinguished from antisense transcripts during processing. It remains to be determined whether the overaccumulation of antisense transcripts has deleterious consequences for dinoflagellate plastid physiology, as in plants (Hotto et al. 2015; Sharwood et al. 2011; Zghidi-Abouzid et al. 2011). However, our data overall provide insights into the diversity and modes of processing associated with transcripts in this unusual plastid lineage. More detailed investigation of the *Karenia mikimotoi* plastid transcriptome may provide valuable insights into the processes that underpin plastid gene expression across the eukaryotes.

## Electronic supplementary material

Supplementary material 1 (PDF 2920 kb)

Supplementary material 2 (PDF 1762 kb)
