# Supplementary material for: Diversity of transcripts and transcript processing forms in plastids of the dinoflagellate alga Karenia mikimotoi
Source: Plant Mol Biol. 2016 Jan 14;90:233–47. doi: 10.1007/s11103-015-0408-9 (PMC4717168; doi:10.1007/s11103-015-0408-9)
Supplement: Supplementary file 1 — Supplementary material 1 (PDF 2920 kb) [file 11103_2015_408_MOESM1_ESM.pdf]

Diversity of transcripts and transcript processing forms in plastids of the dinoflagellate alga *Karenia mikimotoi*

Richard G. Dorrell[1,2], George A. Hinksman[1] and Christopher J. Howe[1]*

[1]Department of Biochemistry, University of Cambridge

[2]School of Biology, École Normale Supérieure, Paris

***to whom correspondence should be addressed: ch26@cam.ac.uk**

Supplementary Figures

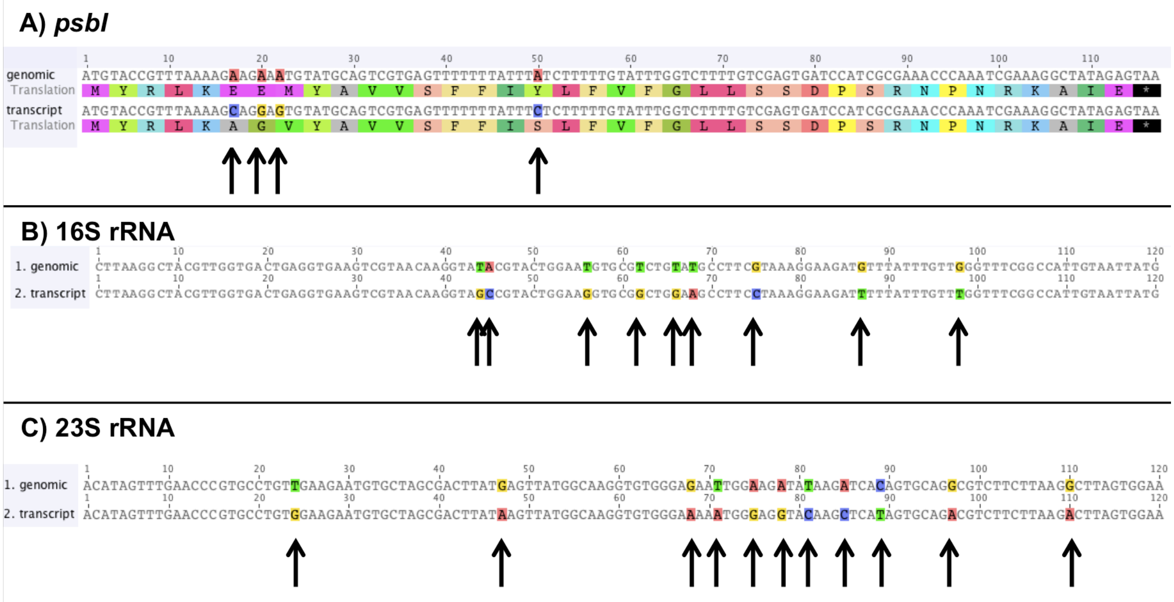

Fig. S1. Presence of editing on *K. mikimotoi psbI* and rRNA transcripts*.* This figure shows the aligned genomic and transcript sequences for the complete CDS of *K. mikimotoi psbI* (**panel A**), and 120 bp regions of 16S (**panel B**) and 23S ribosomal RNA (**panel C**). For *psbI*, the conceptual translation products of the genomic and transcript sequences are also shown. Discrepancies between the genetic and transcript sequences, labelled with arrows, indicate that each transcript is subject to editing.

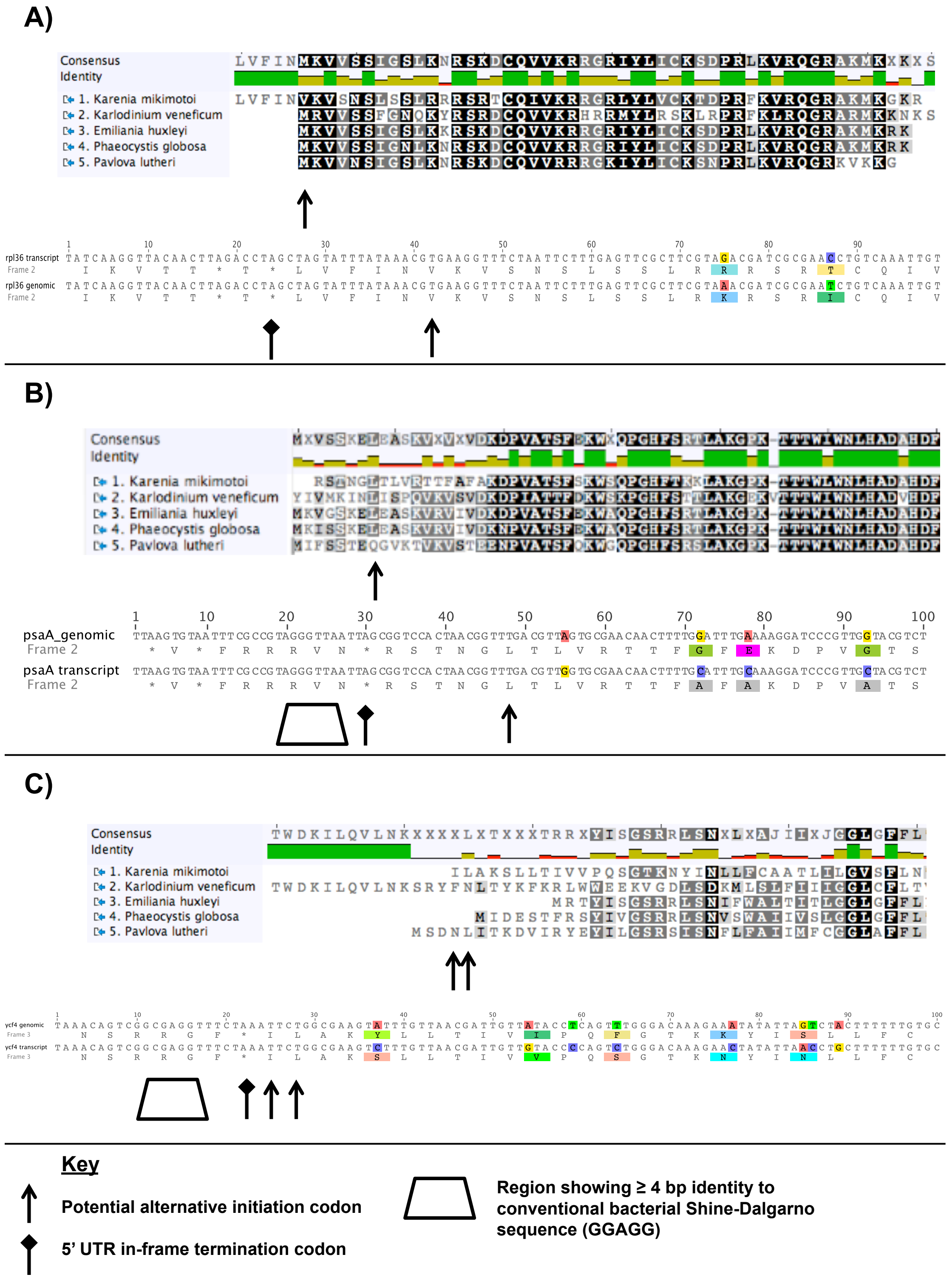
A)
Consensus
Identity
1. Karenia mikimotoi
2. Karlodinium veneficum
3. Emiliania huxleyi
4. Phaeocystis globosa
5. Pavlova lutheri
rpl36 transcript
Frame 2
rpl36 genomic
Frame 2
B)
Consensus
Identity
1. Karenia mikimotoi
2. Karlodinium veneficum
3. Emiliania huxleyi
4. Phaeocystis globosa
5. Pavlova lutheri
psaA_genomic
Frame 2
psaA transcript
Frame 2
C)
Consensus
Identity
1. Karenia mikimotoi
2. Karlodinium veneficum
3. Emiliania huxleyi
4. Phaeocystis globosa
5. Pavlova lutheri
ycf4 genomic
Frame 3
ycf4 transcript
Frame 3
Key
Potential alternative initiation codon
5' UTR in-frame termination codon
Region showing ≥ 4 bp identity to conventional bacterial Shine-Dalgarno sequence (GGAGG)

Fig. S2. Alternative translation initiation codons in *Karenia mikimotoi.*
This figure shows evidence for the use of non-ATG translation initiation codons in the *K. mikimotoi rpl36* (**panel A**), *psaA* (**panel B**) and *ycf4* genes (**panel C**). At the top of each panel, a protein alignment is given for the predicted translation products of the transcript sequence for each gene, along with the orthologous protein sequences inferred from the plastid genomes of the related fucoxanthin dinoflagellate *Karlodinium veneficum,* and the free-living haptophytes *Emiliania huxleyi, Phaeocystis globosa,* and *Pavlova lutheri* (Baurain et al., 2010; Gabrielsen et al., 2011; Puerta et al., 2005). In each case, the *K. mikimotoi* sequence is not predicted to encode a methionine at the consensus N-terminus position found in the haptophyte sequences.

At the bottom of each panel, a nucleotide alignment is given for the genomic (identified by TAiL-PCR) and transcript sequences (identified by circular RT-PCR) for each gene. In each case, the 5’ UTR of the gene is found to contain an in-frame termination codon, immediately upstream of the 5’ end of the conserved coding sequence (labeled with square arrows), eliminating the possibility that translation is initiated from an ATG codon positioned further upstream. Instead, the predicted 5’ end of each coding sequence contains codons recognised to function as alternative translation initiation sites (labeled with open arrows) by NCBI ORF finder, which in the case of *psaA* and *ycf4* are positioned close to sequences with predicted Shine-Dalgarno function. While predicted the *rpl36* initiation codon was not adjacent to a predicted Shine-Dalgarno sequence, no putative Shine-Dalgarno sequences could be found within the entire 5' UTR of this transcript (extending to the 5' end consensus cleavage site identified by circular RT-PCR). Thus, *rpl36, psaA* and *ycf4* transcripts in the *K. mikimotoi* plastid are translated from alternative initiation sites to ATG.

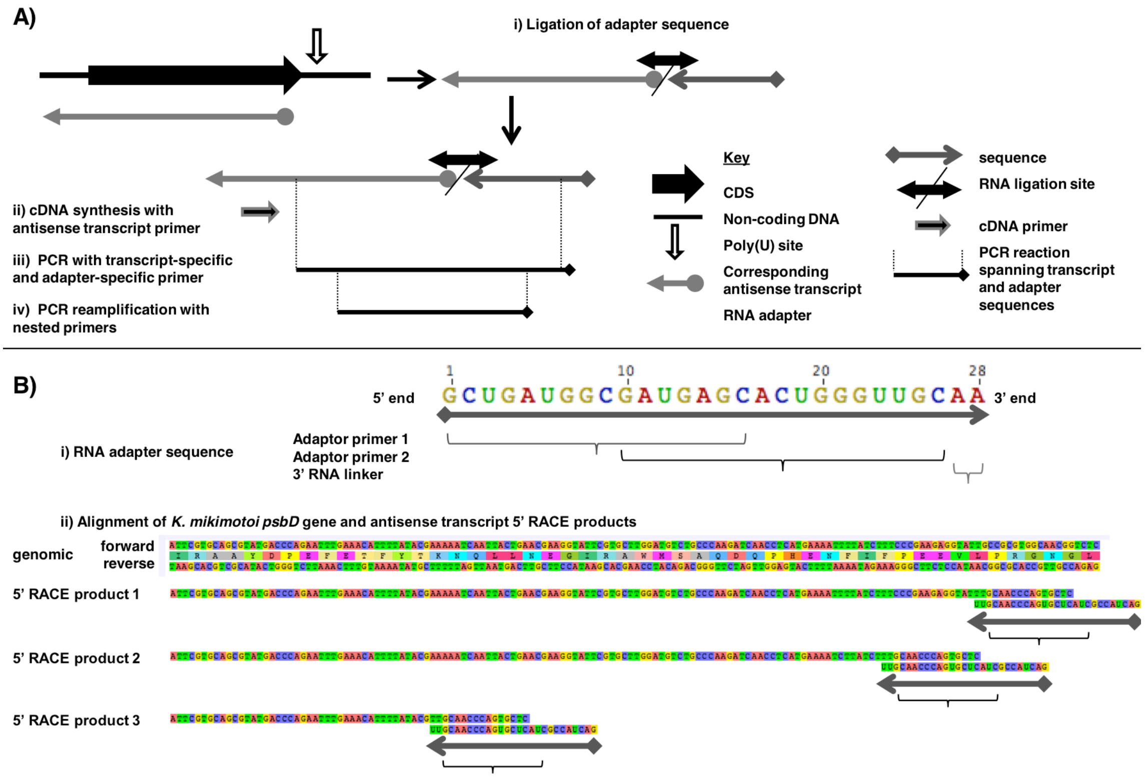

Fig. S3. 5' RACE of *Karenia mikimotoi* plastid antisense transcripts.

Panel A shows a diagram of RNA ligase-mediated 5' RACE. An RNA adapter is ligated to a sample of total cellular RNA. The 3' end of the RNA adapter ligates to the 5' ends of processed transcript sequences. RT-PCR is performed using a cDNA synthesis primer with the same sequence as the non-template strand of minicircle sequence, which will anneal to antisense transcripts. PCR is performed with the cDNA template, a nested PCR primer specific to the antisense transcript sequence, and a forward directed primer specific to RNA adapter sequence. This will amplify antisense transcripts onto which an RNA adapter has been ligated at the 5' end. A second amplification is performed using the initial PCR product, and nested transcript-specific and adapter-specific primers. Products of the second PCR are cloned and sequenced.

(figure legend continued overleaf)

Fig. S3 (continued)

Panel B shows the RNA adapter sequence used for 5’ RACE **(i)**, and the regions of this sequence that correspond to the first and second adaptor-specific primers used. Below this, an alignment is shown of the 3’ end of the *K. mikimotoi psbD* gene, and three sequences obtained using the 5’ RACE protocol using primers specific to antisense *psbD* transcripts **(ii)**. Each sequence aligns with the *psbD* template strand, followed by a region that aligns with the 3’ end of the RNA adapter used (shown below each product in reverse complemented form). The six bp of the *K. mikimotoi* plastid genome sequences located immediately downstream of each adaptor ligation site (Ligation product 1: TGCCGC; Ligation product 2: TTCCCG; Ligation product 3: AAAAAT) are not complementary to the 3’ end of the second adaptor-specific PCR primer used (sequence: GGTTGC; complement: GCAACC), indicating that they are not the result of promiscuous annealing of the adaptor-specific primer to unligated sense transcripts. Each ligation site thus demarcates the 5’ terminus of a transcript sequence, and given the orientation of these termini relative to the *psbD* CDS, they must be derived from antisense transcripts.

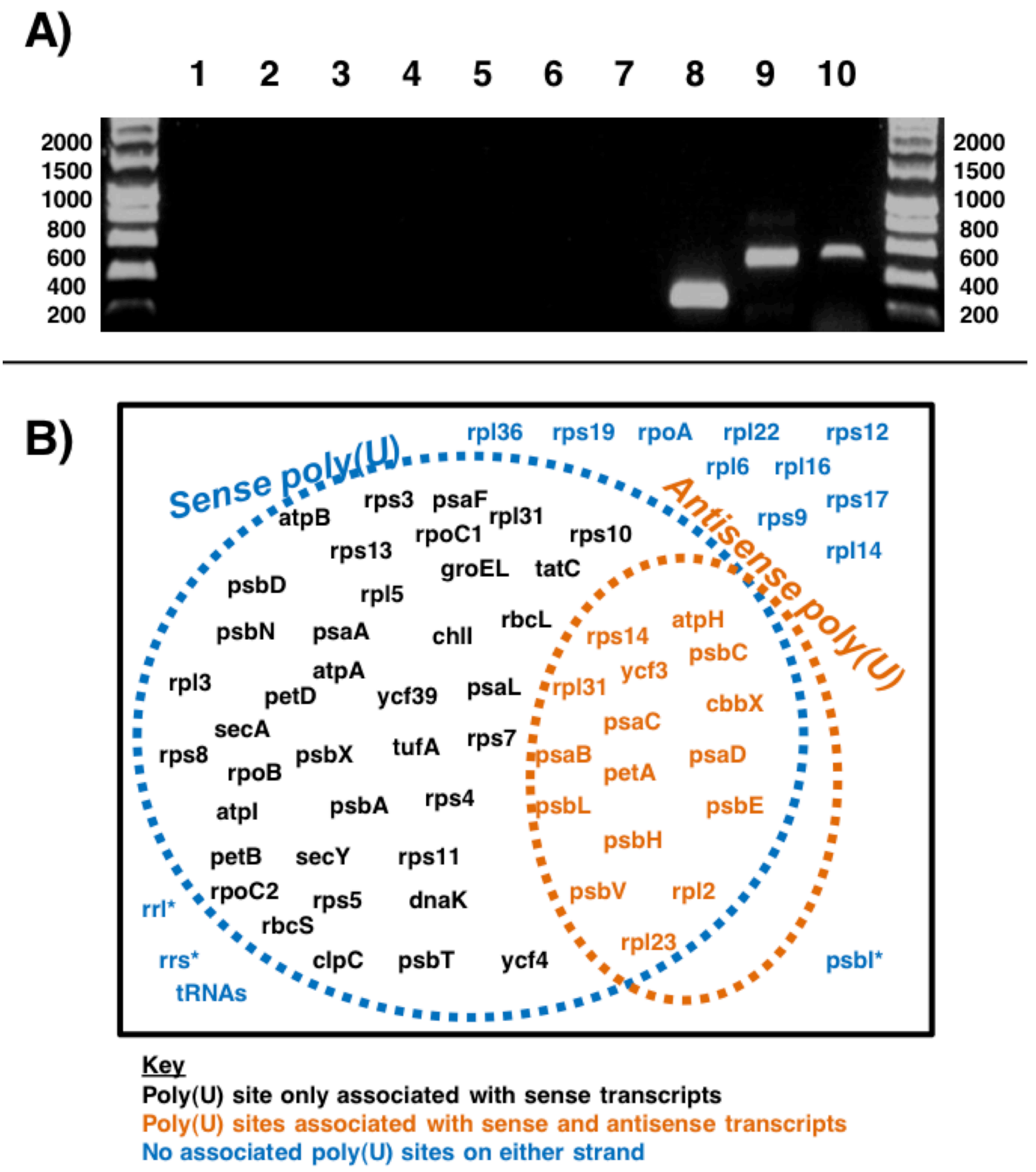

Fig. S4: Absence of poly(U) tails from antisense *K. mikimotoi* plastid transcripts. **Panel A** shows the gel photograph of a series of RT-PCRs performed with oligo-d(A) cDNA to detect polyuridylylated sense and antisense transcripts of seven genes (*psbA, psbD, psaA, rbcL, ycf4, rps13, rps11*) in the *Karenia mikimotoi* plastid. Lanes **1-7**: PCRs performed with an oligo-d(A) cDNA template and PCR primer, and PCR primers with the same sequence as the template strands of seven genes (*psbA, psbD, psaA, rbcL, ycf4, rps13, rps11*), indicating that polyuridylylated antisense transcripts are absent. Lanes **8-10**: RT-PCRs performed with oligo-d(A) cDNA and an oligo-d(A) PCR primer together with PCR primers with the same sequence as the non-template strands of three genes (*psbA, psbD, rps13*),confirming the presence of polyuridylylated sense transcripts.

(figure legend continued overleaf)

Fig. S4 (continued)

Panel B shows the number of genes identified across the entire *K. mikimotoi* plastid to give rise to polyuridylylated sense and antisense transcripts. Genes are colour-coded according to the identification of poly(U) sites associated with sense or antisense transcripts. Genes marked with an asterisk are ones that did not possess a poly(U) site in the associated 3’ UTR of the non-template strand, but were identified as part of a polyuridylylated polycistronic sense transcript.
