# Supplementary material for: Diversity of transcripts and transcript processing forms in plastids of the dinoflagellate alga Karenia mikimotoi
Source: Plant Mol Biol. 2016 Jan 14;90:233–47. doi: 10.1007/s11103-015-0408-9 (PMC4717168; doi:10.1007/s11103-015-0408-9)
Supplement: Supplementary file 2 — Supplementary material 2 (PDF 1762 kb) [file 11103_2015_408_MOESM2_ESM.pdf]

Diversity of transcripts and transcript processing forms in plastids of the dinoflagellate alga *Karenia mikimotoi*

Richard G. Dorrell[1,2], George A. Hinksman[1] and Christopher J. Howe[1]*

[1]Department of Biochemistry, University of Cambridge

[2]School of Biology, École Normale Supérieure, Paris

***to whom correspondence should be addressed: ch26@cam.ac.uk**

Supplementary Tables

Table S1. Primers used for oligo-d(A) RT-PCR of *K. mikimotoi* plastid NGS contigs

oligo-d(A) GGGACTAGTCTCGAGAAAAAAAAAAAAAAAAAA

Gene	PCR forward primer	Gene	PCR forward primer
**atpA**	GTGATTTAAGTGCCTTTTTACCC	**rpl14**	CAGGTTGATTCGGCAAC
**atpB**	GGATTAATGGGATATCAACCG	**rpl16**	AAAATGAGTGGAAATTTATTCATG
**atpH**	GGGACATTATTACTTTCATTAGCC	**rpl2**	GTCCGACTGGTATTTAAAAGTTG
**atpI**	GGGCCAACTACCTAATTCG	**rpl22**	CAAACCCCGTCTCAAAGG
**cbbX**	CATACGGCACCCAGAAC	**rpl23**	GTTTCGCGGTAGCGTAG
**chlI**	GACGCAGTAAAAACCGC	**rpl3**	CGTCCCGCAGATAGACATC
**clpC**	CACCACCGGGTTATGTTG	**rpl31**	GATGGGCAACTTATTTTGAAAG
**dnaK**	TCTCGGCACGACAAATAG	**rpl36**	CTTTTAGGATAAAATATCAAGGTTACAAC
**groEL**	GCGACCGAGACTGAGATG	**rpl5**	TTCAGACGTGCCTTTCC
**petA**	GACCCATCAGACGAGCAAC	**rpl6**	CCTACCGGAATAGAAGTGACTG
**petB**	TTGTCAGGGCTGGATG	**rpoA**	TGCTAGAACAACTCCCGC
**petD**	TGTCTACGGCTTTCCTGC	**rpoB**	TGGTCGTACTGGACTTGC
**psaA**	GCCGGTCTAGTTCTAGCAG	**rpoC1**	AATGGTATTCCTGTTGGAGAG
**psaB**	TCTCAAAGGGGAGGGG	**rpoC2**	CGGGCGCCAACTTTAC
**psaC**	CGTGTCCTTGTGATGTTCTTG	**rps10**	TGCGTTCTTATAGTCCAGCC
**psaD**	GTGGTTACGTGGGTTGC	**rps11**	TTAGCAATACAATTGCAACACTTAC
**psaF**	CGCGATCTGTATAGGTTGG	**rps12**	GGTTCGAGGAGGTCGTG
**psaL**	GGCTGCACCTATTTCGAG	**rps13**	GCTCTCGAAAACGGAAATC
**psbA**	GCTATCAGGCTCACTTTTATATGC	**rps14**	CTCGTAATTCTGCACCGAC
**psbB**	ATCGCGGCAGGAACTC	**rps17**	CAGGTTGATTCGGCAAC
**psbC**	CGACGGCTGCTGAAG	**rps19**	GGTCACGGAGCTCAACTATAC
**psbD**	TATCAGTGGGAGGTTGGTTAAC	**rps3**	TGAGATTCGTCGGCAG
**psbE**	AACAGGCGAACGTCCG	**rps4**	CTGCAGGCTAAACAACGAC
**psbH**	CAGCAGGTTCTCCCGAC	**rps5**	CTACTAACACGCTCACGCTC
**psbI-1**	AAGGCTCATGATTTTGGG	**rps7**	GGTTCGAGGAGGTCGTG
**psbI-2**	GCTTAGCTAACGGATTTACAATC	**rps8**	TTCAGACGTGCCTTTCC
**psbI-3**	TCAGGGAAGGTTGATGGTAAAG	**secA**	GGTATGGGCAAAAGTAAACC
**psbL**	AGATAACCCATTCGAAGGC	**secY**	TTGGGCTTTATTGCTGC
**psbN**	CGAGTTCTCTACATATAGCTGGTG	**tatC**	CAGGTCAACTAAGCGGG
**psbT**	TCTTACTTTTTGGTACTCTAGGGG	**tufa**	AGTTCCAGAAGCGGACG
**psbV**	TTCTGAGTTACACCCTTGTCC	**ycf3**	GTCTCTCAGCCCAAACATC
**psbX**	CTTCATGCAAAGTCTCGTTC	**ycf39**	CAACAATACTAGTGGCCGG
**rbcL**	GCGGAGTTAGAAAGCCC	**ycf4**	CAGAATTCATACCTCAAGGGTTAG
**rbcS**	CTCATGATCCGCACCTTC		

Table S2: Tabulated plastid transcript sequences from *Karenia mikimotoi*.

Gene	Extent	Read coverage	Contig length (bp)	Poly(U) transcript?	Poly(U) site in 3' UTR?	3' UTR length (bp)	Poly(U) length (bp)
**atpA**	5' Partial	171.29	876	Y	Y	16	22
**atpB**	5' Partial	99.20	783	Y	Y	29	28
**atpH**	Complete	16.08	336	Y	Y	5	54
**atpI**	5' Partial	12.98	336	Y	Y	21	34
**cbbX**	Complete	5.86	1005	Y	Y	13	21
**chlI**	5' Partial	20.44	772	Y	Y	3	23
**clpC**	5' Partial	2.88	1150	Y	Y	18	19
**dnaK**	5' Partial	11.65	1363	Y	Y	9	16
**groEL**	5' Partial	5.92	522	Y	Y	12	28
**petA**	5' Partial	12.69	905	Y	Y	27	22
**petB**	5' Partial	45.95	511	Y	Y	7	29
**petD**	5' Partial	18.26	497	Y	Y	10	23
**psaA**	Complete	165.94	2383	Y	Y	21	24
**psaB**	Complete	96.31	2369	Y	Y	10	21
**psaC**	Complete	7.58	313	Y	Y	20	28
**psaD**	Complete	7.89	652	Y	Y	2	33
**psaF**	Complete	11.91	590	Y	Y	28	23
**psaL**	5' Partial	2.09	284	Y	Y	5	23
**psbA**	Complete	3442.63	1127	Y	Y	21	20
**psbB**	5' Partial	44.74	606	Y	Y	0	18
**psbC**	Complete	123.42	1489	Y	Y	11	20
**psbD**	Complete	257.93	1066	Y	Y	9	6
**psbE**	Complete	3.06	374	Y	Y	6	37
**psbH**	Complete	9.66	389	Y	Y	6 to 28	30
**psbI**	Complete	5.93	445	N	N	x	x
**psbL**	Complete	1.91	212	Y	Y	4	37
**psbN**	Complete	3.12	373	Y	Y	32 to 104	29
**psbT**	5' Partial	1.93	164	Y	Y	6	83
**psbV**	Complete	12.88	545	Y	Y	3	24
**psbX**	5' Partial	2.05	356	Y	Y	3	22
**rbcL**	5' Partial	237.82	1326	Y	Y	7	23
**rbcS**	5' Partial	312.49	439	Y	Y	4	99
**rpl14**	Complete	6.52	1559	Y	Y	21	44
**rpl16**	Complete	6.00	409	Y	N	x	x
**rpl2**	Complete	5.80	1033	Y	Y	40	62
**rpl22**	Complete	9.69	1507	Y	N	x	x
**rpl23**	Complete	7.24	493	Y	Y	20	65
**rpl3**	Complete	1.51	718	Y	Y	23	12
**rpl31**	Complete	5.92	1770	Y	N	x	24
**rpl36**	Complete	0.40	1475	Y	N	x	x
**rpl5**	Complete	8.72	1559	Y	Y	8	36
**rpl6**	5' Partial	32.95	895	Y	N	x	x
**rpoA**	5' Partial	4.00	717	Y	Y	33	24
**rpoB**	5' Partial	9.78	494	Y	Y	20	25
**rpoC1**	5' Partial	4.68	1009	Y	Y	3	26
**rpoC2**	5' Partial	2.51	575	Y	Y	4	94
**rps10**	5' Partial	6.29	490	Y	Y	50	59
**rps11**	Complete	3.97	1475	Y	Y	31	12
**rps12**	Complete	9.15	1770	Y	Y	59	x
**rps13**	Complete	5.96	1475	Y	Y	27	15
**rps14**	5' Partial	2.15	362	Y	Y	18	33
**rps17**	5' Partial	7.44	1559	Y	N	x	x
**rps19**	5' Partial	2.76	1507	Y	N	x	x

Table S2 (continued)

Gene	Extent	Read coverage	Contig length (bp)	Poly(U) transcript?	3' UTR poly(U) site?	3' UTR length (bp)	Poly(U) length
**rps3**	Complete	16.90	1507	Y	Y	99	27
**rps4**	5' Partial	13.60	471	Y	Y	49	25
**rps5**	Complete	12.33	895	Y	Y	55	31
**rps7**	Complete	15.03	1770	Y	Y	3	25
**rps8**	Complete	13.79	1559	Y	Y	43	22
**rps9**	5' Partial	6.81	1770	Y	N	x	x
**rrl**	Internal	3298.86	767	N	N	x	x
**rrs**	Complete	2381.44	1304	N	N	x	x
**secA**	5' Partial	6.15	1446	Y	Y	37	24
**secY**	Complete	4.83	1025	Y	Y	54	19
**tatC**	5' Partial	12.24	683	Y	Y	25	21
**tufA**	5' Partial	70.32	327	Y	Y	3	32
**ycf3**	Complete	2.29	556	Y	Y	5	23
**ycf39**	Complete	7.07	1061	Y	Y	20	21
**ycf4**	Complete	9.46	772	Y	Y	3	13
**Total**			63322				
**Average**		185.7				19.7	30.4

Table S3. Gene clusters identified in *Karenia mikimotoi*

This table lists the gene clusters identified in *K. mikimotoi* via the direct assembly of transcriptome data, and via thermal asymmetric interlaced PCR. Primers for the thermal asymmetric interlaced PCRs that yielded multigene contigs are listed at the bottom. (anti) denotes a gene in a reverse transcriptional orientation relative to the remainder of the contig.

1. Gene clusters	Method of assembly	Poly(U) genes
psbC-tRNA^{Met}	TAiL-PCR	psbC
psbD-tRNA^{Met}-ycf4-(anti)rpoA	TAiL-PCR	psbD; ycf4; rpoA
rbcL-tRNA^{Phe}	TAiL-PCR	rbcL
rpl16-rps17-rpl14-rpl5-rps8	Assembled from transcriptome data	rpl14; rpl5; rps8
rpl31-rps12-rps7	Assembled from transcriptome data	rps12; rps7
rpl36-rps13-rps11-(anti)atpI	Assembled from transcriptome data (rpl36-rps13-rps11) and TAiL-PCR (atpI)	rps13; rps11; atpI
rpl6-rps5	Assembled from transcriptome data	rps5
rps19-rpl22-rps3	Assembled from transcriptome data	rps3
(anti)tRNA^{Tyr}-psbI	TAiL-PCR	none
tufA-psaA	TAiL-PCR	tufA; psaA

2. Primers for TAiL-PCR

Contig	gene-specific primer 1	gene-specific primer 2
psbC-tRNA^{Met}	AATAGATGATTACTAGTAATAAATATAAAGAGGC	TAATCAACAACATTTTTAATTTAATCG
psbD-tRNA^{Met}-ycf4	GCTATTCACGGAGCGAC	CAAACGGTGGTTACACTTCTTC
ycf4-(anti)rpoA	AAAACTAACGGTACATAATTATGCTAGAC	GCTCAGTTAGCCAATGGG
rbcL-TRNAPhe	CAACGATACTCCAGATGATCAAC	CCGCTAATAAAAATAGAAACTTATCC
rrps11-(anti)atpI	TTAGCAATACAATTGCAACACTTAC	ACGAGGTGGAATACTAAAGAGG
(anti)tRNA^{Tyr}-psbI	AACATACCTTACTCTATAGCCTTTCG	TTTGGGTTTCGCGATG
tufA-psaA	CTAGCGGAATCAAATAAACGAC	CACGTTGTGCCAATTCC

Contig	gene-specific primer 3		Arbitrary degenerate primers
psbC-tRNA^{Met}	GCTACTTCCTTTAACTTTGAGGC	1	TTNTCGASTWTSGWGTT
psbD-tRNA^{Met}-ycf4	TGGTAATGGTCTCTAACACGTC	2	TTWGTGNAGWANCANAGA
ycf4-(anti)rpoA	TGTAATCTCGAAGTCCTCG	3	CCTTNTWGAWTWTWGWWTT
rbcL-tRNA^{Phe}	CCCTTTCTAAATTTTTAGAGTCG	4	CCTTWGTGNAWWANCANAWA
rps11-(anti)atpI	CCGTCGAAGACAACATTCTTAG	5	GGAACWACNTWTWNGTNTTW
(anti)tRNA^{Tyr}-psbI	GTAGGGAAGCAGGTGTTGG	6	TTACWACANGWWGNTGNTWT
tufA-psaA	CGACAAAAGACCAAATACAAAAAG	7	GGAANACTWAWAWCWWAWA
		8	TTAANCWAGWCWCWAWWAA

Table S4: Genes of probable plastid origin identified from *Karlodinium veneficum* nuclear EST libraries.

This table lists contigs assembled from sequences identified *Karlodinium veneficum* nuclear ESTs by reciprocal tBLASTn/ BLASTx searches with *Karenia mikimotoi* plastid transcript sequences. The complete protein sequences associated with each contig are shown, alongside protein targeting predictions obtained using SignalP-3.0 and ASAfind (Bendtsen *et al.*, 2004; Gruber *et al.*, 2015). The SignalP D score, and ASAfind 20 aa transit peptide score for each protein are given in brackets.

	Coverage	Constituent accessions
**1. psaD**	Complete	AmSd244SL1 , Am2d85SL1, CAMNT0009143429, CAMNT0009184911, CAMNT0009236345, CAMNT0009337407, CAMNT0009338495, CAMNT0009366831, CAMNT0009433183, CAMNT0009435019, CAMNT0009477167
**Sequence**	MRALVFAVATLSHAALAASMKSRTVPENLDRLAALLFAQTGVPASASTSATRAQGARMEEWMPDRTGQMFIRDGEVEKYVM<br>TWTSKNEQIIELPTGGAASMKSGENLMYFRKKEQALALSRYLKTNFKIADFKVYRIYPGGEVQYIHPADGVPSEKVNAGRIGVG<br>NVPWSIGKNPRVGKFEESNPTNKFGNWRYRNQGPLID*	
**Targeting**	SignalP postivie (**0.79),** ALA-AS SP cleavage site, followed by cTP (**1.43**)	
**2. psaL**	Complete	CAMNT_0009446733, CAMNT_0009168921, CAMNT_0009319011, CAMNT_0009299771, CAMNT_0009186299, CAMNT_0009327403, CAMNT_0009158107, CAMNT_0009237257, CAMNT_0009480477, CAMNT_0009469005, CAMNT_0009476275
**Sequence**	MQGQAEVGKSKFGRLIGDENKMGIWGTNPLIVSYLEGLPIYRENISPFMRGLEIGMAHGYWVPGPFTIGGPLRNSVDCVQAGA<br>FSAAMMGVILTLGGCTAYGIAQGFDQDDDKSKLKGGGWFDFRNGAFIGWMGGAIVYFLAEKYLLGKTGF*	
**Targeting**	SignalP negative (**0.05**)	
**3. rpl22**	Complete	KME00004684, KME00008386, CAMNT_0009515829, CAMNT_0009453053, CAMNT_0009328289, CAMNT_0009269149, CAMNT_0009384017, CAMNT_0009478777, CAMNT_0009237119, CAMNT_0009162807, CAMNT_0009329549
**Sequence**	MWRTSMIVAHLASSILAFSPPLSYRAGETSSGVAMRRLADALMKSGNNRIRDPSIAGYAKARNVRMSPTKVRRPINEIRGKSYAE<br>ALTLLEYMPYHSCMPIAKVVKSAAANAVNNHGYDNIADLYV?AAYVDQGPTLKRMRPRAQGRAYSIQKKTCSITIEMKEKEKPKE<br>EA*	
**Targeting**	SignalP postivie (**0.72),** ILA-FS SP cleavage site, followed by cTP (**5.48**)	
**4. rpl23**	Complete	AmSd316SL1, CAMNT_0009328399, CAMNT_0009374763, CAMNT_0009505843, CAMNT_0009171989, CAMNT_0009151439, CAMNT_0009238193, CAMNT_0009316109, CAMNT_0009268657, CAMNT_0009468403, CAMNT_0009383861, CAMNT_0009322553
**Sequence**	MALRVLVSIALACLAREAHTENEETEKLASLLFALVPQHPQMKVATSGQPVMKARTHLKPPPKKGNPRQPRETYYRNNPILDYD<br>LIKYPVLTEKSIKNIENHQTYTFAVARDADKPEIKAAIEGLFNVSVKKLNTLNAPPKRRRVGKTTGKARQYKRAFVRVKEGDSITLF<br>EEE*	
**Targeting**	SignalP positive (**1.00**), AHT-EN SP cleavage site; cTP negative (**1.19**)	
**5. tatC**	Complete	CAMNT_0009441717, CAMNT_0009192201, CAMNT_0009318735
**Sequence**	MRAVIYAVCIGSVCTAWRRTGPSDPLKRRGNVGSAPPSREFETKQSLANVLLAANPALTFRAPAAYSARVDGKGRHREGHRM<br>QISDAVDPESEKPPMPWELEGSNDLSMSLGSHIEEFRERLIFAGMMVVLLILASFGFSKDLINVLKSPIVEQGVKFIQSNPSEYFF<br>TSIKVSGYSGLLLAAPIILNQIIAYVVPGLTASEKKLFGPLLLGSTALFYVGLTFGFGVLGPAALNFFLAFAEESVESFFSIDEYADF<br>VGFMMLSTGVAFQVPIIQTLLAKLGVVNSQQMFDAWRYVVVGAVILAAFLTPSTDPLTQLLLAGPLVGLYLGGAAVVRLTEGEK<br>PKPPSISGGEE*	
**Targeting**	SignalP positive (**0.67**), VCT-AW cleavage site, followed by cTP (**1.14**)	

Table S5. Tabulated indels identified across 9179 aa aligned fucoxanthin dinoflagellate plastid protein sequence

Indels are listed by form (insertion, deletion, and N- and C-terminal extension) and by gene. Indels were identified by alignment against orthologous plastid protein sequences from the haptophytes *Emiliania huxleyi, Phaeocystis globosa,* and *Pavlova lutheri*. Indels were only counted if they were not found in any species other than *Karenia mikimotoi* or *Karlodinium veneficum.*

		Evolutionary distribution		
**1. By form**	**Total**	**Both taxa**	***Karenia mikimotoi***	***Karlodinium veneficum***
**Insertions**	59	8	21	30
**Deletions**	25	0	9	16
**N-terminal extensions**	9	1	4	4
**C-terminal extensions**	16	1	9	6
**Total**	109	10	43	56

		Evolutionary distribution		
**2. By gene**	**Alignment length**	**Both taxa**	***Karenia mikimotoi***	***Karlodinium veneficum***
**atpA**	245	0	0	3
**atpB**	236	0	0	0
**atpH**	61	0	0	1
**atpI**	61	0	0	0
**cbbX**	291	0	4	1
**chlI**	128	0	0	0
**clpC**	275	0	2	0
**dnaK**	443	1	6	0
**groEL**	57	0	0	0
**petA**	284	1	3	3
**petB**	151	0	0	0
**petD**	141	0	1	0
**psaA**	767	0	1	3
**psaB**	468	0	0	3
**psaC**	82	0	1	0
**psaF**	185	2	3	7
**psbA**	206	0	0	0
**psbB**	199	0	0	0
**psbC**	472	0	0	1
**psbD**	199	0	0	0
**psbE**	85	0	2	1
**psbH**	67	0	0	0
**psbI**	39	0	0	0
**psbL**	39	0	1	1
**psbN**	44	1	0	1
**psbT**	28	0	0	0
**psbV**	165	0	1	0
**rbcL**	302	0	0	0
**rbcS**	112	0	0	0
**rpl14**	122	0	0	1
**rpl16**	127	1	1	0
**rpl2**	176	0	0	2
**rpl3**	107	0	0	2

Table S5 (continued)

	Alignment length	Evolutionary distribution		
		Both taxa	*Karenia mikimotoi*	*Karlodinium veneficum*
**rpl31**	71	0	1	0
**rpl36**	49	0	1	0
**rpl5**	98	0	0	0
**rpl6**	85	0	0	0
**rpoA**	191	0	3	2
**rpoC1**	283	1	1	5
**rps11**	131	0	2	1
**rps12**	90	0	0	0
**rps13**	125	0	2	0
**rps14**	49	0	0	1
**rps19**	59	0	0	1
**rps3**	217	0	1	4
**rps4**	77	0	1	0
**rps5**	160	1	0	0
**rps7**	157	1	0	4
**rps8**	140	1	0	2
**secA**	57	0	0	0
**secY**	161	0	1	2
**tufA**	95	0	0	0
**ycf3**	172	0	2	1
**ycf39**	220	0	1	2
**ycf4**	128	0	1	1
**Total**	9179			

Table S6. Primers used for circular RT-PCR of *Karenia mikimotoi* plastid transcripts.

A. 5’ end identification of *psaA, rpl36* and *ycf4* transcripts

Gene	cDNA primer	PCR reverse primer	PCR forward primer
**psaA**	CTAGCGGAATCAAATAAACGAC	CGACAAAAGACCAAATACAAAAAG	GCCGGTCTAGTTCTAGCAG
**rpl36**	CCCTTTTCGTTTTACAATTTG	ATCGTTTACGAAGCGAACTC	GCTCTCGAAAACGGAAATC
**ycf4**	TCTGGAATTGACAGTTGACAG	CGTTAACAAATACTTCGCCAG	GCTCAGTTAGCCAATGGG

B. Transcript mapping of *rpl36-rps13-rps11* and *psbD-tRNA^Met-ycf loci*

**1. rpl36-rps13-rps11**	**rps13**	**rps11**
**cDNA primer 1**	GTTGTCCTCGAGTTGGAAG (rps13 3' end)	GCAATTGTATTGCTAAAGTTAGCTAATATATG (rps11 5' end)
**cDNA primer 2**	CCCTTTTCGTTTTACAATTTG (rps13 5'end)	
**Reverse primer 1**	CCCTTTTCGTTTTACAATTTG (rps13 5' end)	CCCTTTTCGTTTTACAATTTG (rps13 5' end)
**Reverse primer 2**	ATCGTTTACGAAGCGAACTC (rps13 5' end)	ATCGTTTACGAAGCGAACTC (rps13 5' end)
**Reverse primer 3**		GTTGTCCTCGAGTTGGAAG (rps13 3' end)
**Reverse primer 4**		TTTAATTAAATACCTAGGAAATATCAACTGTAAC (rps13 3' end)
**Reverse primer 5**		CTAGGAAATATCAACTGTAACTCTTGC (rps11 5' end)
**Reverse primer 6**		CGAAATCCCTTCCAATTTTG (rps11 5' end)
**Forward primer 1**	GCTCTCGAAAACGGAAATC (rps13 5' end)	
**Forward primer 2**	AACGTTATTGAAGATCCCAAAC (rps13 3' end)	
**Forward primer 3**	CGGAAGCGGTATTAAGGC (rps13 3' end)	
**Forward primer 4**	AAGTTCAAATGAAGTAAGACTCAAAAG (intergenic)	
**Forward primer 5**	TTAGCAATACAATTGCAACACTTAC (rps11 5' end)	
**Forward primer 6**	ACGAGGTGGAATACTAAAGAGG (rps11 3' end)	ACGAGGTGGAATACTAAAGAGG (rps11 3' end)
**Forward primer 7**	CCGTCGAAGACAACATTCTTAG (rps11 3' end)	CCGTCGAAGACAACATTCTTAG (rps11 3' end)

**2. psbD-Met^CAT-ycf4**	**psbD**	**ycf4**
**cDNA primer 1**	CCTCCTAGTTCAAGCCACC (psbD 5' end)	TCTGGAATTGACAGTTGACAG
**Reverse primer 1**	AAGTAATCCTGACCAACCAATG (psbD 5' end)	AAGTAATCCTGACCAACCAATG (psbD 5' end)
**Reverse primer 2**	GTGTGGAAACGGCTGC (psbD 5' end)	GTGTGGAAACGGCTGC (psbD 5' end)
**Reverse primer 3**		CCTCCTAGTTCAAGCCACC (psbD 5' end)
**Reverse primer 4**		CAACCGTGCTATTTCAAACTG (psbD 5' end)
**Reverse primer 5**		GTTTTCATGAGGTTGATCTTGG (psbD 3' end)
**Reverse primer 6**		GCGACCTTGGGCTTATG (tRNA^Met)
**Reverse primer 7**		TATATTTCTTTGTCCCAAACTGAG (ycf4 5' end)
**Reverse primer 8**		CGTTAACAAATACTTCGCCAG (ycf4 5' end)
**Forward primer 1**	GCTATTCACGGAGCGAC (psbD 3' end)	
**Forward primer 2**	CAAACGGTGGTTACACTTCTTC (psbD 3' end)	
**Forward primer 3**	TGGTAATGGTCTCTAACACGTC (psbD 3' end)	
**Forward primer 4**	CATAAGCCCAAGGTCGC (tRNA^Met)	
**Forward primer 5**	CGTTCAATCTTCTCCTCAAC (tRNA^Met)	
**Forward primer 6**	CAGAATTCATACCTCAAGGGTTAG (ycf4 5' end)	
**Forward primer 7**	AAAACTAACGGTACATAATTATGCTAGAC (ycf4 3' end)	AAAACTAACGGTACATAATTATGCTAGAC (ycf4 3' end)
**Forward primer 8**	GCTCAGTTAGCCAATGGG (ycf4 3' end)	GCTCAGTTAGCCAATGGG (ycf4 3' end)
**Forward primer 9**	TCTGGAATTGACAGTTGACAG (ycf4 3' end)	TCTGGAATTGACAGTTGACAG (ycf4 3' end)
**Forward primer 10**	TTGACAGCTGACAACTAAATTAGTG (ycf4 3' end)	TTGACAGCTGACAACTAAATTAGTG (ycf4 3' end)

Table S7. Circular RT-PCR data for the *K. mikimotoi rpl36-rps13-rps11* and *psbD-tRNA^{Met}-ycf4* loci.

This table lists all of the circular RT-PCR products obtained for sense transcripts over each locus, and the PCR primers used to identify them. Sequences with identifical terminus positions were generated by sequencing the ligation products from different PCR reactions, performed with separate batches of cDNA template, hence represent separate transcripts. Terminus positions are given relative to the underlying CDS. PCR primer numbers correspond to those given in Table S6. Transcripts of a length equivalent to bands identified in northern blots, and that contain complete open reading frames (i.e. may correspond to the principal translated mRNAs of the particular gene) are highlighted in bold, and the corresponding band number is listed as per Fig. 2.

	Transcript dimensions				Primers		Northern band	Notes
	5' end	3' end	Poly(U)	Length	R	F		
**rpl36-rps13**								
**Non-poly(U) transcript 1**	**-29**	**21**	**0**	**676**	**2**	**1**	**Ii**	
**Non-poly(U) transcript 2**	**-29**	**54**	**0**	**709**	**2**	**3**	**Ii**	**3' end extends into rps11**
**Non-poly(U) transcript 3**	**-29**	**87**	**0**	**742**	**2**	**2**	**Ii**	**3' end extends into rps11**
**Non-poly(U) transcript 4**	**-29**	**87**	**0**	**742**	**2**	**2**	**Ii**	**3' end extends into rps11**
**Non-poly(U) transcript 5**	**-29**	**103**	**0**	**758**	**2**	**3**	**Ii**	**3' end extends into rps11**
**Non-poly(U) transcript 6**	**-29**	**41**	**0**	**696**	**2**	**1**	**Ii**	
Non-poly(U) transcript 7	-29	183	0	838	2	2		3' end extends into rps11
**Non-poly(U) transcript 8**	**-28**	**129**	**0**	**783**	**2**	**2**	**Ii**	**3' end extends into rps11**
**Non-poly(U) transcript 9**	**-28**	**129**	**0**	**783**	**2**	**2**	**Ii**	**3' end extends into rps11**
**Non-poly(U) transcript 10**	**-28**	**129**	**0**	**783**	**2**	**2**	**Ii**	**3' end extends into rps11**
**Non-poly(U) transcript 11**	**-25**	**129**	**0**	**780**	**2**	**2**	**Ii**	**3' end extends into rps11**
**Non-poly(U) transcript 12**	**-25**	**129**	**0**	**780**	**2**	**2**	**Ii**	**3' end extends into rps11**
Non-poly(U) transcript 13	-12	-15	0	623	2	1		
Non-poly(U) transcript 14	-11	-11	0	626	2	1		
Non-poly(U) transcript 15	1	100	0	725	2	2		3' end extends into rps11
Non-poly(U) transcript 16	5	23	0	644	2	1		
Non-poly(U) transcript 17	5	-170	0	451	2	2		
Non-poly(U) transcript 18	13	-194	0	419	2	2		
Non-poly(U) transcript 19	22	14	0	618	2	3		
Non-poly(U) transcript 20	22	32	0	636	2	2		
Non-poly(U) transcript 21	27	160	0	759	2	2		3' end extends into rps11
**Poly(U) transcript 1**	**-30**	**126**	**11**	**782**	**2**	**3**	**Ii**	**Poly(U) site situated in *rps11* CDS**
**Poly(U) transcript 2**	**-30**	**126**	**11**	**782**	**2**	**3**	**Ii**	**Poly(U) site situated in *rps11* CDS**
**Poly(U) transcript 3**	**-29**	**32**	**5**	**687**	**2**	**2**	**Ii**	
**Poly(U) transcript 4**	**-29**	**38**	**17**	**693**	**2**	**3**	**Ii**	
**Poly(U) transcript 5**	**-29**	**87**	**5**	**742**	**2**	**3**	**Ii**	**Poly(U) site situated in *rps11* CDS**
**Poly(U) transcript 6**	**-29**	**34**	**10**	**689**	**2**	**1**	**Ii**	
**Poly(U) transcript 7**	**-29**	**38**	**13**	**693**	**2**	**1**	**ii**	**Poly(U) tail contains single A**
**Poly(U) transcript 8**	**-29**	**38**	**13**	**693**	**2**	**3**	**ii**	
**Poly(U) transcript 9**	**-25**	**34**	**14**	**699**	**2**	**2**	**ii**	
**rps11**								
Non-poly(U) transcript 1	-115	-8	0	838	4	6		5' end extends into rps13
poly(U) transcript 1	-50	30	8	819	4	6		5' end extends into rps13
poly(U) transcript 2	52	31	12	722	6	7		
poly(U) transcript 3	52	31	12	722	6	7		

Table S7 (continued).

	Transcript dimensions				Primers		Northern band	Notes
	5' end	3' end	Poly(U)	Length	R	F		
**rpl36-rps13-rps11**								
Non-poly(U) transcript 1	-29	-313	0	1118	2	6		
**Poly(U) transcript 1**	**-29**	**112**	**4**	**1547**	**2**	**8**	**i**	
**Poly(U) transcript 2**	**-29**	**30**	**8**	**1469**	**2**	**8**	**i**	
**Poly(U) transcript 3**	**-29**	**31**	**10**	**1472**	**2**	**8**	**i**	
**Poly(U) transcript 4**	**-29**	**31**	**11**	**1473**	**2**	**8**	**i**	
**Poly(U) transcript 5**	**-29**	**31**	**12**	**1474**	**2**	**5**	**i**	
**Poly(U) transcript 6**	**-29**	**31**	**13**	**1475**	**2**	**5**	**i**	
**Poly(U) transcript 7**	**-29**	**30**	**14**	**1475**	**2**	**6**	**i**	
**Poly(U) transcript 8**	**-29**	**31**	**14**	**1476**	**2**	**8**	**i**	
**Poly(U) transcript 9**	**-29**	**30**	**14**	**1475**	**2**	**8**	**i**	
**Poly(U) transcript 10**	**-29**	**31**	**15**	**1477**	**2**	**8**	**i**	
**Poly(U) transcript 11**	**-29**	**31**	**16**	**1478**	**2**	**6**	**i**	
**Poly(U) transcript 12**	**-29**	**31**	**17**	**1479**	**2**	**6**	**i**	**Poly(U) tail contains single A**
**Poly(U) transcript 13**	**-29**	**31**	**17**	**1479**	**2**	**8**	**i**	
**Poly(U) transcript 14**	**-29**	**31**	**18**	**1480**	**2**	**8**	**i**	
**Poly(U) transcript 15**	**-29**	**30**	**21**	**1482**	**2**	**6**	**i**	
Poly(U) transcript 16	22	31	21	1432	2	8		
**psbD**								
Non-poly(U) transcript 1	-139	278	0	1415	2	4		3' end extends through poly(U) site and $tRNA^{Met}$ into ycf4
Non-poly(U) transcript 2	-132	130	0	1260	1	3		3' end extends through poly(U) site and $tRNA^{Met}$
Non-poly(U) transcript 3	-131	43	0	1172	2	4		3' end extends through poly(U) site into $tRNA^{Met}$
Non-poly(U) transcript 4	-129	92	0	1219	2	4		3' end extends through poly(U) site into $tRNA^{Met}$
**Non-poly(U) transcript 5**	**-74**	**8**	**0**	**1080**	**1**	**3**	**i**	
Non-poly(U) transcript 6	-69	-171	0	896	2	3		
**Non-poly(U) transcript 7**	**-60**	**7**	**0**	**1065**	**2**	**3**	**i**	
Non-poly(U) transcript 8	-42	-28	0	1012	2	3		
Non-poly(U) transcript 9	-30	-100	0	928	2	3		
Non-poly(U) transcript 10	6	-88	0	904	2	3		
Non-poly(U) transcript 11	22	-178	0	798	2	3		
Non-poly(U) transcript 12	24	-33	0	941	2	3		
Non-poly(U) transcript 13	74	72	0	996	2	3		
Non-poly(U) transcript 14	129	40	0	909	2	3		3' end extends through poly(U) site
**Poly(U) transcript 1**	**-120**	**12**	**8**	**1130**	**1**	**3**	**i**	
**Poly(U) transcript 2**	**-118**	**11**	**9**	**1127**	**1**	**3**	**i**	
**Poly(U) transcript 3**	**-118**	**12**	**8**	**1128**	**1**	**3**	**i**	
**Poly(U) transcript 4**	**-53**	**10**	**1**	**1061**	**2**	**3**	**i**	
**Poly(U) transcript 5**	**-53**	**10**	**1**	**1061**	**2**	**3**	**i**	
**Poly(U) transcript 6**	**-5**	**12**	**6**	**1015**	**2**	**3**	**i**	
**psbD-$tRNA^{Met}$-ycf4**								
Non-poly(U) transcript 1	6	118	0	2036	2	7		

Table S7 (continued).

	Transcript dimensions				Primers		Northern band	Notes
	5' end	3' end	Poly(U)	Length	R	F		
**ycf4**								
Non-poly(U) transcript 1	-191	274	0	1128	7	9		5' end extends into $tRNA^{Met}$; 3' end extends through poly(U) site
Non-poly(U) transcript 2	-191	274	0	1128	7	9		5' end extends into $tRNA^{Met}$; 3' end extends through poly(U) site
Non-poly(U) transcript 3	-191	274	0	1128	7	9		5' end extends into $tRNA^{Met}$; 3' end extends through poly(U) site
Non-poly(U) transcript 4	-191	274	0	1128	7	9		5' end extends into $tRNA^{Met}$; 3' end extends through poly(U) site
Non-poly(U) transcript 5	-146	-141	0	668	8	8		
Non-poly(U) transcript 6	-130	-248	0	545	8	8		
Non-poly(U) transcript 7	-129	-179	0	613	8	8		
Non-poly(U) transcript 8	-105	-233	0	535	7	7		
Non-poly(U) transcript 9	-47	-195	0	515	8	8		
Non-poly(U) transcript 10	-29	-56	0	636	7	7		
Non-poly(U) transcript 11	-25	-55	0	633	8	8		
Non-poly(U) transcript 12	-21	-56	0	628	7	7		
Non-poly(U) transcript 13	-21	-56	0	628	7	7		
Non-poly(U) transcript 14	-21	-56	0	628	7	7		
Non-poly(U) transcript 15	-20	-4	0	679	7	9		
Non-poly(U) transcript 16	5	-76	0	582	8	8		
**Poly(U) transcript 1**	**-105**	**-2**	**12**	**766**	**7**	**9**	**ii**	
**Poly(U) transcript 2**	**-105**	**3**	**12**	**771**	**7**	**9**	**ii**	
**Poly(U) transcript 3**	**-105**	**4**	**12**	**772**	**7**	**9**	**ii**	

Table S8. Northern blot probes to detect *Karenia mikimotoi* plastid transcripts.

This table lists the sequence of the T7 arm of the pGEM-T Easy vector alongside the first 50 bp of each probe sequence complementary to *K. mikimotoi* plastid gene sequences. The range of sequence covered by each probe is given relative to the underlying CDS, as identified by PCR.

T7 arm TAATACGACTCACTATAGGGCGAATTGGGCCCGACGTCGCATGCTCCCGGCCGCCATGGCCGCGGGATT

Probe	start	end	Sequence
**rpl36**	129	10	GTCCTTGCCGAACTTTAAATCGTGGATTTGTCTTACAAACTAGGTAAAAT...
**rps13**	402	303	GTTGTCCTCGAGTTGGAAGACCCGCGTTGCGTCTTTTTCCCCTCAGGGTT...
**rps11**	466	232	CCCCAACCTGCACCAGCTACAGTCACTTGTACCTCAGTTATGTTGAACAT...
**psbD**	393	70	CTATAGGACCAGAAAAAGCAATCGCATTGTAAGGTCTAATACCGACCAAC...
**ycf4**	665	493	TTGACCAATTCGCATATAATATTTTACACTAATTTAGTTGTCAACTGTCA...

Table S9. Primers used to sequence non-polyuridylylated and polycistronic transcripts from the rpl36-rps13-rps11 and psbD-tRNA^{Met}-ycf4 loci

Where a PCR reverse primer sequence is not specifically provided, the cDNA synthesis primer was used as the PCR reverse primer.

Transcript	cDNA synthesis/ PCR reverse primer	PCR forward primer
**Poly(U) psbD-tRNA^{Met}-ycf4**	cDNA primer: GGGACTAGTCTCGAGAAAAAAAAAAAAAAAAAA<br>reverse primer: GACCAATTCGCATATAATATTTTAC	TATCAGTGGGAGGTTGGTTAAC
**Non-poly(U) rpl36-rps13**	CGAAATCCCTTCCAATTTTG	GCTCTCGAAAACGGAAATC
**Non-poly(U) rpl36-rps13-rps11**	GCTTTTTTTAAAGATGACTGCG	GCTCTCGAAAACGGAAATC
**Non-poly(U) rps11**	GCTTTTTTTAAAGATGACTGCG	TTAGCAATACAATTGCAACACTTAC
**Non-poly(U) psbD**	GCGACCTTGGGCTTATG	TATCAGTGGGAGGTTGGTTAAC
**Non-poly(U) ycf4**	CTTAAAAGCTAACGTAATGAAACTTC	CAGAATTCATACCTCAAGGGTTAG
**Non-poly(U) psbD-tRNA^{Met}-ycf4**	CTTAAAAGCTAACGTAATGAAACTTC	TATCAGTGGGAGGTTGGTTAAC

Table S10. Primers for RT-PCRs to detect antisense plastid transcripts in *Karenia mikimotoi*.

This table lists the primers used to (i) identify antisense transcripts and (ii) confirm specificity of the antisense transcript cDNA primers used. Sense cDNA template sequences (used as positive controls for the cDNA specificity tests) were generated using Primer (2) of the corresponding gene.

i. cDNA synthesis primers

Gene	Primer 1- Antisense transcripts	Sense transcript cDNA primer
psbA	GCTATCAGGCTCACTTTTATATGC	CCATCGTAGAAACTCCCATAG
psbD	TATCAGTGGGAGGTTGGTTAAC	GTTTTCATGAGGTTGATCTTGG
psaA	CACGTAGTTCAGCTCTGATACC	CACGTTGTGCCAATTCC
rbcL	GATGCGTATGGCAGGTG	GTTGATCATCTGGAGTATCGTTG
ycf4	CAGAATTCATACCTCAAGGGTTAG	GACCAATTCGCATATAATATTTTAC
rps13	GCTCTCGAAAACGGAAATC	GTTGTCCTCGAGTTGGAAG
rps11	TTAGCAATACAATTGCAACACTTAC	CCCCAACCTGCACCAG

ii. Primers to confirm the specificity of the antisense cDNA synthesis primer

	Primer 2- upstream PCR forward primer	Primer 3- PCR reverse primer
psbA	ATCACAGCAGACAACACCCG	TACCCCCATTGTAAAGCC
psbD	ACGACTGGCTAAAACGAGAC	AAAATATTAGCTATGTTTATTCAAGTACAAC
psaA	GCCGGTCTAGTTCTAGCAG	CACGTTGTGCCAATTCC
rbcL	GCGGAGTTAGAAAGCCC	GTTGATCATCTGGAGTATCGTTG
ycf4	TGGTAATGGTCTCTAACACGTC	GACCAATTCGCATATAATATTTTAC
rps13	CTTTTAGGATAAAATATCAAGGTTACAAC	GTTGTCCTCGAGTTGGAAG
rps11	ATCGTTTACGAAGCGAACTC	CCCCAACCTGCACCAG

Table S11. Primers used for 5' RACE of *Karenia mikimotoi* antisense transcripts.

This table lists the primers used to amplify 5' ends of *Karenia mikimotoi* antisense transcripts by RNA-ligase mediated 5' RACE. Antisense transcript 5' ends for each gene were amplified using cDNA synthesis primer A, in conjunction with gene-specific PCR primers 1 and 2 (primer combination A), and using cDNA synthesis primer B, in conjunction with gene-specific PCR primers 2 and 3 (primer combination B).

**RNA adapter**	GCUGAUGGCGAUGAGCACUGGGUUGCAA	
**Adapter-specific PCR primer 1**	GCTGATGGCGATAGC	
**Adapter-specific PCR primer 2**	GATGAGCACTGGGTTGC	
**Gene**	**rps13**	**rps11**
**cDNA synthesis primer A**	GCTCTCGAAAACGGAAATC	AAGTTCAAATGAAGTAAGACTCAAAAG
**cDNA synthesis primer B**	CCCTTTTCGTTTTACAATTTG	TTAGCAATACAATTGCAACACTTAC
**Gene-specific PCR primer 1**	CCCTTTTCGTTTTACAATTTG	TTAGCAATACAATTGCAACACTTAC
**Gene-specific PCR primer 2**	ATCGTTTACGAAGCGAACTC	ACGAGGTGGAATACTAAAGAGG
**Gene-specific PCR primer 3**	GTTGTCCTCGAGTTGGAAG	CCGTCGAAGACAACATTCTTAG
**Gene**	**psbD**	**ycf4**
**cDNA synthesis primer A**	TATCAGTGGGAGGTTGGTTAAC	CGTTCAATCTTCTCCTCAAC
**cDNA synthesis primer B**	TTGAACTAGGAGGCTTGTGG	CAGAATTCATACCTCAAGGGTTAG
**Gene-specific PCR primer 1**	TTGAACTAGGAGGCTTGTGG	CAGAATTCATACCTCAAGGGTTAG
**Gene-specific PCR primer 2**	GCTATTCACGGAGCGAC	AAAACTAACGGTACATAATTATGCTAGAC
**Gene-specific PCR primer 3**	CAAACGGTGGTTACACTTCTTC	GCTCAGTTAGCCAATGGG

Table S12. Antisense *rpl36-rps13-rps11* and *psbD-tRNA^{Met}-ycf4* transcript termini as identified by circular RT-PCR and 5' RACE.

This table lists the 5' and 3' termini of antisense transcripts from the *rpl36-rps13-rps11* and *psbD-tRNA^{Met}-ycf4* loci, as per Table S7. The very limited editing events observed for each antisense transcript 5’ RACE product are also listed.

The terminus positions of the antisense transcripts are shown relative to the terminus positions of the corresponding CDS. Note that as the antisense transcripts are in opposing orientation to the CDS, the antisense transcript 5' terminus is given relative to the 3' terminus of the CDS, and vice versa. The PCR primers used to amplify 5' RACE products are listed in Table S11; primers used to identify each circular RT-PCR product correspond to those listed in Table S6.

**1. rpl36-rps13-rps11**	**5' end**	**Position**	**Length**	**Editing events**	**Primer Combination**	**Notes**
Antisense rps11 transcript 5' end 1	2050	311	715	0	A	Extends through poly(U) site
Antisense rps11 transcript 5' end 2	1996	257	331	0	B	Extends through poly(U) site
Antisense rps11 transcript 5' end 3	1990	251	325	0	B	Extends through poly(U) site
Antisense rps11 transcript 5' end 4	1960	221	243	5	B	Extends through poly(U) site
Antisense rps11 transcript 5' end 5	1762	23	428	0	A	Terminates just before poly(U) site
Antisense rps11 transcript 5' end 6	1676	-67	341	3	A	
Antisense rps11 transcript 5' end 7	1598	-141	263	0	A	
**2. psbD-tRNAMet-ycf4**	**5' end**	**Position**	**Length**	**Editing events**	**Primer Combination**	**Notes**
Antisense psbD transcript 5' end 1	In CDS	-19	248	0	B	
Antisense psbD transcript 5' end 2	In CDS	-33	233	1	A	
Antisense psbD transcript 5' end 3	In CDS	-108	160	0	A	
Antisense psbD transcript 5' end 4	In CDS	-465	211	0	A	
Antisense ycf4 transcript 5' end 1	In UTR	69	244	0	B	Extends through poly(U) site
Antisense ycf4 transcript 5' end 2	In CDS	-21	150	2	B	
Antisense ycf4 transcript 5' end 3	In CDS	-53	119	0	B	
Antisense ycf4 transcript 5' end 4	In CDS	-110	321	2	A	
Antisense ycf4 transcript 5' end 5	In CDS	-267	164	1	A	

Table S12 (continued)

B. Circular RT-PCR

1. rpl36-rps13-rps11	Transcript dimensions 5' end	3' end	Poly(U)	Length	PCR primers R	F	Notes
**rps13**							
Antisense transcript 1	14	-51	0	618	F3	R2	3' end extends into rpl36
Antisense transcript 2	14	-51	0	618	F3	R2	3' end extends into rpl36
**rps11**							
Antisense transcript 1	19	-497	0	953	F6	R3	3' end extends through rps13 poly(U) site
Antisense transcript 2	19	-497	0	953	F6	R3	3' end extends through rps13 poly(U) site
Antisense transcript 3	19	-493	0	957	F6	R3	3' end extends through rps13 poly(U) site
Antisense transcript 4	19	-493	0	957	F6	R3	3' end extends through rps13 poly(U) site

2. psbD-tRNA^{Met}-ycf4	Transcript dimensions 5' end	3' end	Poly(U)	Length	PCR primers R	F	Notes
**psbD**							
Antisense transcript 1	316	22	0	1292	F1	R2	5' end extends through poly(U) site into ycf4
Antisense transcript 2	-12	149	0	837	F1	R2	
Antisense transcript 3	-34	-124	0	1088	F1	R2	
Antisense transcript 4	-45	103	0	850	F1	R2	
Antisense transcript 5	-94	45	0	859	F1	R2	
Antisense transcript 6	-100	-25	0	923	F1	R2	
**ycf4**							
Antisense transcript 1	-56	-17	0	624	F7	R8	
Antisense transcript 2	-56	-21	0	628	F8	R8	
Antisense transcript 3	-56	-21	0	628	F7	R8	
Antisense transcript 4	-56	-21	0	628	F7	R8	

Table S13. Primers for RT-PCRs to detect polyuridylylated antisense transcripts.

Primers used for the RT-PCRs shown in Panel A of Fig. S4 are shown in bold text.

oligo-d(A) GGGACTAGTCTCGAGAAAAAAAAAAAAAAAAAA

Antisense transcript poly(U) tests

Gene	PCR reverse primer	Gene	PCR reverse primer
atpA	GAAGAAGCATGTCGTCGC	rpl16	GTAATTTATGCGAAGCTAATCG
atpB	CGCAGGGACGTATATTGC	rpl2	CCCCAATGCAACTTTACC
atpH	ACACAATGCAACAACAAGACC	rpl22	CTGAAGTGCTTTCCCCG
atpI	AGATTCAGCAATGTACGAACAAG	rpl23	CTATTTTCGCATCACCTGC
cbbX	GCCAAATAGCGGACGTAGAG	rpl3	GGCAACGAACCTTTGAGG
chlI	AGAACGGGAGACCTGGG	rpl31	GTCGTGATCCCAACCG
clpC	AAAGCCGGGGTGAGTAAG	rpl36	GTAAAGTCGCCCTCTTCG
dnaK	GCATCCAATGTAGCCCG	rpl5	CGCTGATGACGACGAG
groEL	GTAGACGCATCGTAGCCAC	rpl6	CGAAGCAAAGTGACCTACCC
petA	GCAGAAGGCGTACCTAACG	rpoA	GGTAGCGGTTGGAGTTG
petB	TCCACCACGAAGTAACGC	rpoB	GGTATCCCCGGTTTTGG
petD	GCCGAAGCAGAAATCAAC	rpoC1	CCAGTACTCGGCGACC
**psaA**	**GTAGGGAAGCAGGTGTTGG**	rpoC2	CGAACCCAAACGAAGG
psaB	AATGGAACCAACCTGCG	rps10	AAACTGGTCTCGGGAGG
psaC	CCTGCTAATACGCCAGACC	**rps11**	**GCAATTGTATTGCTAAAGTTAGCTAATATATG**
psaD	GAGGCGAGCGCATTC	rps12	CGATCTTTCACCGGCAC
psaF	ACGGTTGTAGAAGCCTTCC	**rps13**	**GTTGTCCTCGAGTTGGAAG**
psaL	TTTGTCACTTTCTGCGTCAG	rps14	CCTAACTACCAACTTGAGCG
**psbA**	**TACCCCCATTGTAAAGCC**	rps17	TCGTGGTTGCGCTTG
psbB	CACCCTTGTGCGATACC	rps19	GTATAGTTGAGCTCCGTGACC
psbC	CCAGCGCCTAGAACGG	rps3	CGTTCCAGTAATTGCGC
**psbD**	**AAAATATTAGCTATGTTTATTCAAGTACAAC**	rps4	GACAAGGCGAACAAAACC
psbE	GCACCAAAACGTTCGG	rps5	AACAAAACCGCTCGTGC
psbH	GTTGATCCCCAGGCAG	rps7	CAGCAGCAGCTACAATCC
psbI	AACATACCTTACTCTATAGCCTTTCG	rps8	CGACCTCCTCCATAGGC
psbL	GTCTGACACACTCTTAGTTCAAAAAAATAAC	secA	AGCTGTCGACTTCGCTCC
psbN	GGAAAGGATCGCGGAG	secY	CCAGCTATCACGACCCC
psbT	TCGCAATTCTTGGGCTATC	tatC	CTTGGACAAAGCAGGGG
psbV	TCCACCCCATTTTTCACC	tufA	CAGTAACAGTCCCAGCCC
psbX	GATCCTATCGAGAGAGCTAACCGG	ycf3	CGTAACCATAACCGCGTG
**rbcL**	**GTTCCCGCATGGATATG**	ycf39	CTCAGACGACGGTGTAGC
rbcS	GGTTCTCCCACGTGCTTC	**ycf4**	**GAGAAATAATCCTAATATTATTCCGATG**
rpl14	TTCGCGGTGTGCTTG		

Sense positive controls

Gene	PCR forward primer
**psbA**	**GCTATCAGGCTCACTTTTATATGC**
**rps11**	**TTAGCAATACAATTGCAACACTTAC**
**ycf4**	**CAGAATTCATACCTCAAGGGTTAG**
